# Spatial-temporal evolution of technology flows in China’s Beijing-Tianjin-Hebei region: Patent transfer networks 2003–2021

**DOI:** 10.1371/journal.pone.0301509

**Published:** 2024-06-27

**Authors:** Xumei Yuan, Fuli Wei, Ming Zhang, Xu Zhang

**Affiliations:** 1 School of Economics and Management, Yanshan University, Qinhuangdao, China; 2 Beijing-Tianjin-Hebei Synergistic Development Management Innovation Research Center, Yanshan University, Qinhuangdao, China; Sichuan University, CHINA

## Abstract

This paper presents new evidence on knowledge flows in the Beijing-Tianjin-Hebei region of China, involving 43 cities (districts) in the Beijing-Tianjin-Hebei region, based on the invention patent transfer data from the State Intellectual Property Office of China. First, the characteristics of technology flows in the Beijing-Tianjin-Hebei region are analyzed in terms of changes in the number of flows, types of flowing subjects and spatial distribution characteristics. Then, a multi-level patent technology flow network in the Beijing-Tianjin-Hebei region was constructed, and the structural characteristics and node characteristics of each level network were explored separately. The key findings of the study are as follows. (1) The number of patented technology flows has been growing over time, showing obvious phase characteristics during the study period. As a whole, the intra-city (district) technology flow in the Beijing-Tianjin-Hebei region is higher than the inter-city (district). (2) The multi-level patent technology flow network in the Beijing-Tianjin-Hebei region shows dynamic characteristics, with more and more mobile subjects participating in the patent technology flow network, some network nodes becoming closer to each other, and the trend of small group technology flow increasing significantly. (3) Enterprises are the core hub of the patent technology flow network in Beijing-Tianjin-Hebei region. Individual invention patent technology transfer also occupies a high proportion and the participation of universities and colleges in the patent technology flow network in the Beijing-Tianjin-Hebei region is gradually increasing. (4) Over time, the flow of patent technology in the 43 cities (districts) in the Beijing-Tianjin-Hebei region has gradually become active and no longer relies excessively on a particular city (district) for patent technology transfer.

## 1 Introduction

Innovation is the source of sustained growth in the world economy, and knowledge flow is one of the most essential and fundamental forms of achieving innovation [1]. For regions, continuous innovation can only be accomplished through continuous knowledge flows with other regions.

In 2021, China’s newly revised Law of the People’s Republic of China on Scientific and Technological Progress once again emphasized the need to promote close cooperation among various types of innovation subjects, orderly flow of innovation factors, and sustainable optimization of the innovation ecosystem, so as to enhance the overall effectiveness of the innovation system. The State Council of the Central Committee of the Communist Party of China (CPC) issued the Overall Plan for Comprehensive Reform of Market-based Allocation of Factors in January 2022, emphasizing that for a significant period of time in the future, emphasis should be placed on the smooth flow of factors in a broader context. It can be seen that the orderly flow of innovation factors is very critical to regional innovation, and the smooth flow of technology factors, as the most central component of innovation factors, is crucial to regional innovation.

As the core region of China’s science and technology development and productivity promotion, the Beijing-Tianjin-Hebei region is an essential part of the national development strategic plan, and its economic and scientific development has attracted much attention. The total quantity of innovation resources in Beijing, Tianjin and Hebei is abundant, but the allocation of science and technology innovation resources in the three regions is uneven and the mobility is low [2]. According to statistics, the technology contracts flowing from Beijing to Tianjin and Hebei in 2021 were 5,434, with a turnover of 35.04 billion yuan, accounting for only 8.1% of all technology contracts flowing from Beijing to other provinces and cities, with more than 90% flowing to regions outside of Tianjin and Hebei, especially the Yangtze River Delta(YRD), Guangdong-Hong Kong-Macao Greater Bay Area(GBA), etc. It is evident that the migration of innovation achievements from Beijing to the Beijing-Tianjin-Hebei region is obviously insufficient. In this context, how to promote the flow of innovation factors in the Beijing-Tianjin-Hebei region and play an essential role in promoting regional innovation has become a new issue that needs to be urgently explored.

So far, limited attention has been devoted to the knowledge flow network in the Beijing-Tianjin-Hebei region. From the perspective of patent transfer, it has mostly focused on the characteristics of technology transfer in terms of subjects and objects (Zhang et al., 2017) [3], technology transfer policies (Wang, 2020) [4], and technology transfer network governance (Shi, 2015) [5], but the granularity of the related network research is not fine enough. And the phenomenon of innovation subjects embedded in mobility networks of various geographical scopes and spatial scales has been neglected to some extent. In this paper, we obtain data on invention patent assignments in the Beijing-Tianjin-Hebei region for the period 2003–2021, which enables us to study the characteristics of the intra-city(district) and inter-city(district) invention patent flows in the Beijing-Tianjin-Hebei region. What are the most active types of organizations in patent assignment? Are they enterprises, colleges & universities, or scientific institutions? Which innovation agents and cities (districts) dominate the flow of technology based on the transfer of invention patents? In order to answer these questions, we categorize the innovative subjects involved in patent transfer into two parts: transferor and transferee. In addition, combined with the address information of innovation subjects, we are able to further track the flow of patented technologies at different spatial scales with the help of social network analysis methods. Based on the above background, this paper concentrates on three aspects: (1) the characteristics of patent technology flow in Beijing-Tianjin-Hebei region. (2) the construction of multi-level patent technology flow network that encompasses the overall, intra-city (district), and inter-city (district). (3) the dynamic evolution of multi-level patent technology flow network.

The remaining amount of the paper appears as follows. Section 2 examines the literature on regional innovation network, technology mobility. Section 3 presents the research design, data sources and network construction. Section 4 analyzes the characteristics of patent technology flows in the Beijing-Tianjin-Hebei region. Section 5 examines the dynamic evolution of the multi-level patent technology flow network in the Beijing-Tianjin-Hebei region. Section 6 summarizes the main research findings and sets forward countermeasure suggestions.

## 2 Literature review

### 2.1 Regional innovation network

The study of regional innovation networks began with the introduction of the concept of innovation networks (Freeman, 1991) [6]. An innovation network is a network structure in which firms, governments, academic and research institutions interact with each other within a certain region and establish interconnections through the selection of partners [7]. Regional innovation networks are based on the interaction of subjects and form stable systems through adaptive behavior, revealing the law of knotting in the development of regional innovation systems [8–10].

In order to scientifically explore regional innovation in the era of network society, regional innovation network research has been initiated in economic geography. The accumulation and acquisition of relational data is a key link in the construction and quantitative analysis of regional innovation networks [11]. At present, there are two primary approaches. One is to measure the strength of inter-regional innovation linkages using a modified gravity model [12]. The second is to construct inter-regional knowledge flow or collaborative innovation networks based on data such as patent citations and paper collaboration [13, 14].

On this basis, network structure, evolutionary trajectories, and spatial scales of knowledge spillover have become the focal point of regional innovation network research. For example, Hoekman et al. (2010) [15] explored the impact of spatial distance and geographical boundaries on regional innovation networks using data from 313 regional cooperation papers in 33 European countries from 2000–2017. Ye et al. (2015) [16] used the patent statistics database of the United States Patent and Trademark Office (USPTO) as the basis, and used methods such as k-core and block modeling in social network analysis to construct transnational patent citation networks in the field of electrical and electronic technology in three different periods from 1984–2006, and analyzed the patterns and characteristics of international knowledge flows. Li and Phelps (2017) [17] analyzed the polycentric characteristics and dynamic evolution of knowledge networks in the Yangtze River Delta urban agglomeration at various spatial scales based on the data of collaborative papers from WOS from 2000–2014. Liu et al. (2022) [18] constructed a global knowledge flow network based on the 2002–2016 United States Patent and Trademark Office (USPTO) dataset by combining patent transfer analysis with social network analysis to study global knowledge flow from the perspective of patent transfer, and found that an increasing number of inventors and assignees participated in the global knowledge flow network, with China and India rapidly rising in the list of knowledge creators and users, with the U.S. being the largest net knowledge inflow participant and the U.K. being the largest net knowledge outflow participant, and the small-world effect is increasingly evident in the cross-border knowledge flow network consisting of 35 major players.

### 2.2 Technology flow

Various actors in the regional network are able to realize continuous innovation through the flow of innovative factors such as technology, which in turn enhances regional innovation capacity [19]. Technology flow is a highly abstract generalization of the concepts of technology transfer, technology diffusion, technology introduction, etc. It is a complex process involving many factors such as technology, economy, society, enterprises, information and humanities, based on the participants of the flow behavior, supported by the socio-economic and cultural environment, powered by the technology potential difference, and conditioned by the flow of information and materials [20].

Patent data are extensively used to measure technology flows [21–23]. Because patent data contain a large quantity of useful information, have a long time span and relatively complete and stable data sources, they can better characterize knowledge production, technological change and regional economic development [24–26]. Many existing studies on technology transfers are mostly based on the analysis of patent citations [27–29], patent licensing [30, 31]. In this paper, we divide and arrange them accordingly based on the research level.

1. Regional level. Jeon and Lee (2014) [32] examined the flow of virtualization technologies between various countries (regions) based on patent citation data. on the other hand, Andersen (2017) [33] chose patent cooperation data as an indicator of technology transfers, and analyzed the impact of regional trade agreements on patent cooperation across countries. Gaétan and Florian (2020) [34] investigated the sources of knowledge flows between developed and developing countries using patent data and found that North America is more likely to benefit from reverse knowledge flows than Europe as China emerges as a technology leader.
2. Organizational level. Roach and Cohen (2013) [35] assessed the validity and accuracy of corporate reverse patent citations as a public research knowledge flow measure by using a newly constructed dataset that matched patents to R&D lab-level survey data. Kang and Su (2014) [36] assessed the validity and accuracy of corporate reverse patent citations as a public research knowledge flow measure by analyzing 1976–2012 USPTO patents to assess the level of knowledge diffusion and knowledge flow across industries. Chang et al. (2017) [37] used both patent counts and patent citations to investigate the technological capabilities and technological knowledge flow of R&D portfolios to encourage manufacturers to invest in PV technologies. Keiko et al. (2021) [38] used company patent matching data from Japanese manufacturing multinationals to examine the feasibility of allocating more R&D activities allocation to country-industries with greater intensity of knowledge flows would improve innovation performance of MNCs.
3. Technology level. Lai et al. (2007) [39] measured the technology flow among seven industries including computer hardware, computer software, communication finance, and e-commerce. Chu and Su (2015) [40] understood the knowledge flow among different types of knowledge owners as assignees of patented technology by identifying the inventor location of the first assignee type. Jiang et al. (2019) [41] used patent citations as indicator of knowledge flow, examined the impact of firms’ global patent social networks on the knowledge flow of business method software patents and found that firms in a relatively central position or located within the same structural equivalent cluster had more citations to their counterparts’ patents, and that knowledge transfer was positively facilitated when both the citing and cited firms performed the roles of rover and gatekeeper/representative, while the gatekeeper/representative companies (alone) cite fewer patents from companies that do not perform such roles. Giglio et al. (2021) [42] propose a method to study cross-country creativity/knowledge flows by analyzing patent citation networks using the aircraft, aviation and aerospace (AAC) industry as a case study. Smojver et al. (2021) [43] identify technology life cycle by performing a dynamic analysis of patent citation networks to stages, mapping patent co-citation networks from patent citation networks, and applying link prediction algorithms to patent co-citation networks to explore knowledge flows within technology domains.

### 2.3 Current research status

In recent years, domestic and foreign economic geographers have conducted in-depth research on regional innovation networks and achieved a series of valuable research results. However, research cases mostly focus on innovation networks led by multinational corporations in developed countries, while research on innovation networks led by local enterprises in developing countries and regions lacks attention. There are numerous studies on intra-city(district) or inter-city(district) innovation networks at a single spatial scale, but there is insufficient research on the evolution laws of innovation networks at multiple spatial scales. We argue that based on different spatial couplings, analyzing the evolutionary process and mechanism of the innovation network, and systematically portraying the pattern and structure of the innovation network from the perspective of time-space topology should be emphasized.

For research on technology flow, scholars at home and abroad have mostly focused on the cooperative R&D perspective. However, the purpose of cooperative R&D is to reduce the difficulty of technical knowledge production rather than to enhance the value of knowledge, and in fact, most of the patents cannot be transformed and applied in the end. In addition, the cooperative R&D of patented technology does not clarify the specific flow trajectory of knowledge among innovation subjects, which makes the innovation network based on patented technology not have directional characteristics. We argue that both cooperative R&D and transfer of patented technology are a form of innovative knowledge flow, but they are in two stages of the innovation chain respectively. The former belongs to the knowledge production stage and the latter belongs to the knowledge application stage. The transfer of patented technology has obvious directional characteristics, while only technical knowledge with application value can be accepted by the transferee of patented technology. Therefore, a re-examination of the structural characteristics and evolutionary patterns of regional innovation networks from the perspective of patent transfers can be attempted in the future.

## 3 Research design, data sources and network construction

### 3.1 Research design

**Fig 1:**
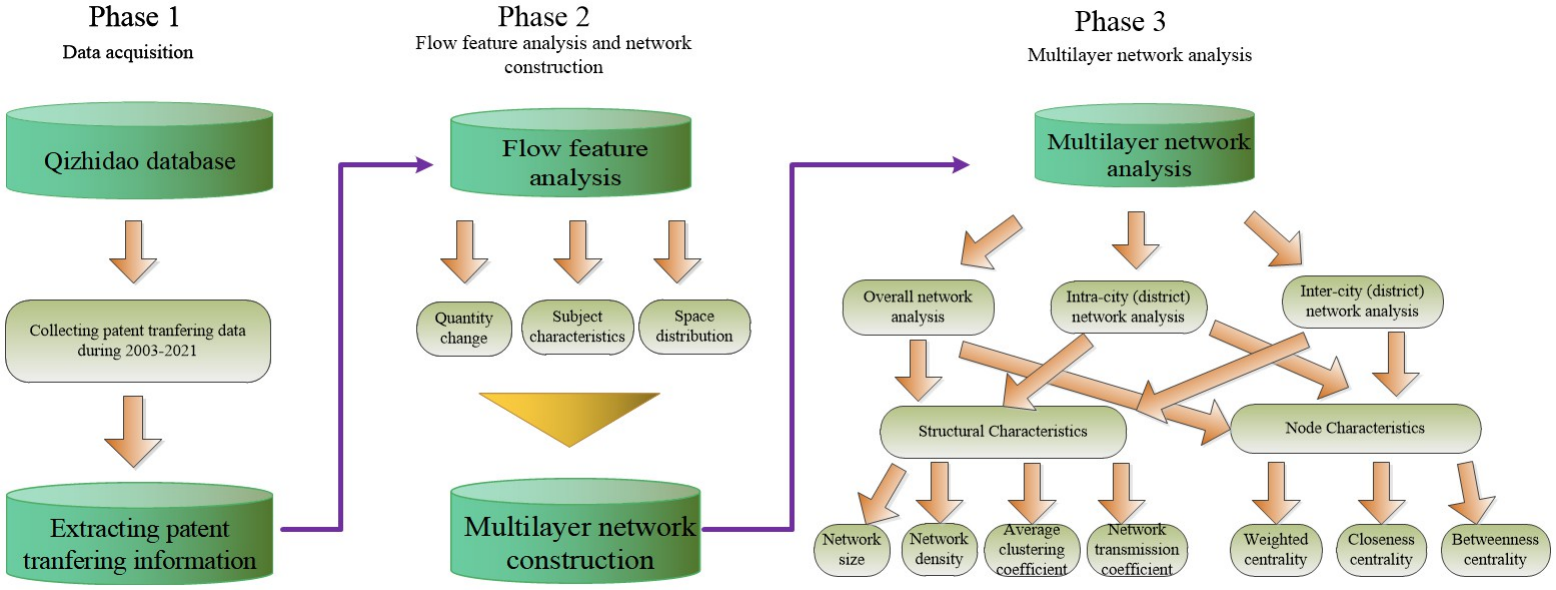
Research flow chart.

The research object of this paper is 43 cities (districts) in the Beijing-Tianjin-Hebei region, each of which is treated as a separate region. The research framework of this paper is shown in Fig 1. Firstly, the data of invention patent transfer in 43 cities (districts) in the Beijing-Tianjin-Hebei region with all the transfer (transferee) organization addresses are collected, and the transfer execution year is set as the time node. Then, the characteristics of technology flow in the Beijing-Tianjin-Hebei region are analyzed in three aspects of flow quantity change, flow subject type and spatial distribution characteristics, and a multi-level patent technology flow network in the Beijing-Tianjin-Hebei region is constructed. Finally, the structural characteristics and node characteristics of each level of network are investigated separately.

### 3.2 Data sources

The invention patent data used in this paper comes from the State Intellectual Property Office of China, on the basis of which we utilize the Qizhidao (https://patents.qizhidao.com/) and Qichacha (https://www.qcc.com/) data platform for searching, cleaning, processing and other work of patent data, the specific steps are as follows. First of all, Searching for transferred patents (only invention patents are considered) from the search service of the patent database of Qizhidao (https://patents.qizhidao.com/), each patent transfer data includes information such as application number, public number, title, application date, announcement date, transferor, transferee, transfer execution date, and number of transfers. Then, relying on the enterprise data platform Qichacha (https://www.qcc.com/), the address information of the transferor (transferee) is searched and identified one by one to ensure that the transferor (transferee) is located in the cities (districts) of Beijing, Tianjin and Hebei. After searching and downloading the patent data, we finally obtained 47,572 invention patent assignments within the Beijing-Tianjin-Hebei region from 2003 to 2021, excluding 47,084 invention patent assignments in which both parties of the assignee and transferee were individuals, involving 15,672 organizations.

### 3.3 Network construction

**Fig 2:**
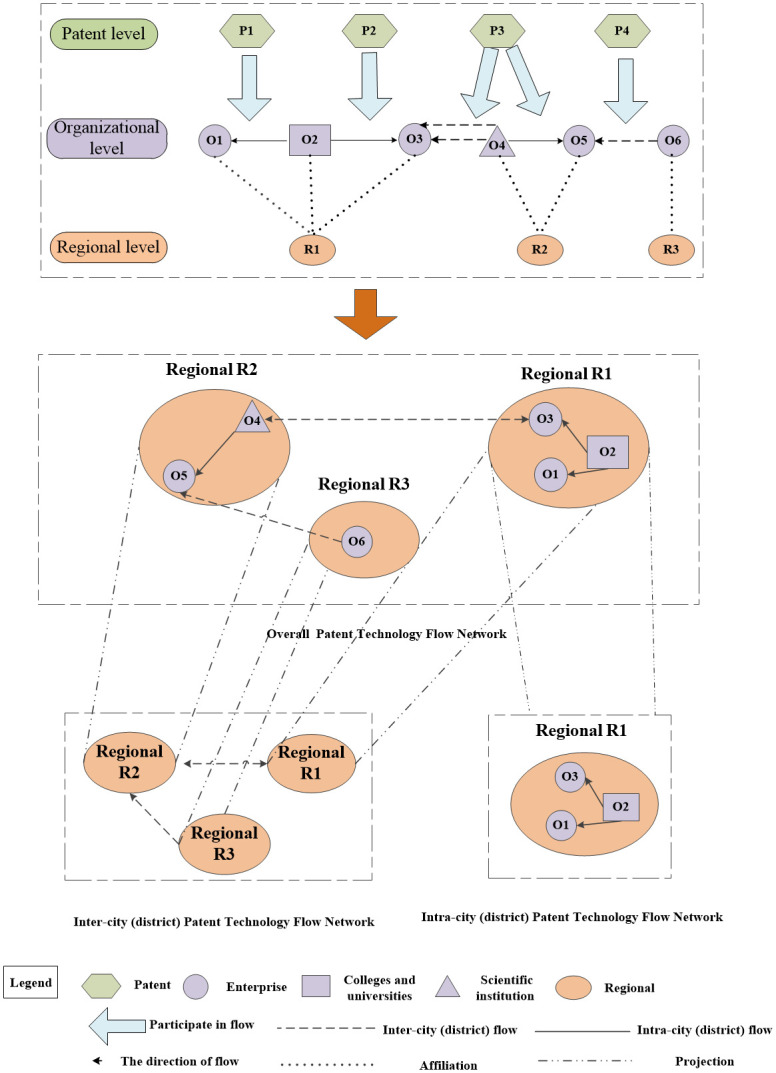
Multi-level patent technology flow network diagram based on patent technology transfer.

The fundamental model of multi-level patent technology flow network is shown in Fig 2. Assuming that there are four transferred invention patents P1, P2, P3 and P4, the organizations concerned are six, O1, O2, O3, O4, O5 and O6, among which four enterprises, one college and university and one scientific institution are distributed in region R1, region R2 and region R3. The transfer relationships among the organizations involved can be expressed as six pairs of network relationships, which are O2→O1, O2→O3, O3→O4, O4→O3, O4→O5, O6→O5.

The transfer relationship between organizations crosses city (district) boundaries and establishes a multi-level patent technology flow network, which is dismantled to include intra-city (district) patent technology flow network and inter-city (district) patent technology flow network. When the assignor and the assignee of a patent for invention are located in the same city (district), such as O2→O1, the intra-city (district) patent technology flow relationship is formed. When the assignor and assignee of a patent for an invention are located in distinct cities (districts), such as O6→O5, the inter-city (district) patent technology flow relationship is formed. The intra-city (district) patent technology flow network is a network formed by the transfer of invention patents between organizations in the same city (district), when the nodes of the network are organizations located in the city (district), and the edge is the patent transfer relationship between organizations in the city (district). The inter-city (district) patent technology flow network consists of the transfer relationship between organizations across regional boundaries, at which time the network node is a city (district), and the edge is the transfer relationship of invention patents formed between cities (districts).

This paper employs the date of execution of invention patent assignment to determine the time when the network nodes establish relationships. This paper assesses the patent technology flow network based on three levels of invention patent assignment: (1) the overall patent technology flow network composed of assignment relationships among all flow subjects. (2) the intra-city (district) patent technology flow network composed of assignment relationships among flow subjects located in the same city (district). (3) the inter-city (district) patent technology flow network composed of assignment relationships among flow subjects spanning different cities (districts), with 43 cities (districts) in Beijing, Tianjin and Hebei, including 16 municipal districts in Beijing, 16 municipal districts in Tianjin and 11 municipalities in Hebei as network nodes, and the transfer relationship between cities (districts) as inter-node links.

## 4 Characteristics of the Beijing-Tianjin-Hebei region’s patent technology flow

### 4.1 Quantity change

**Fig 3:**
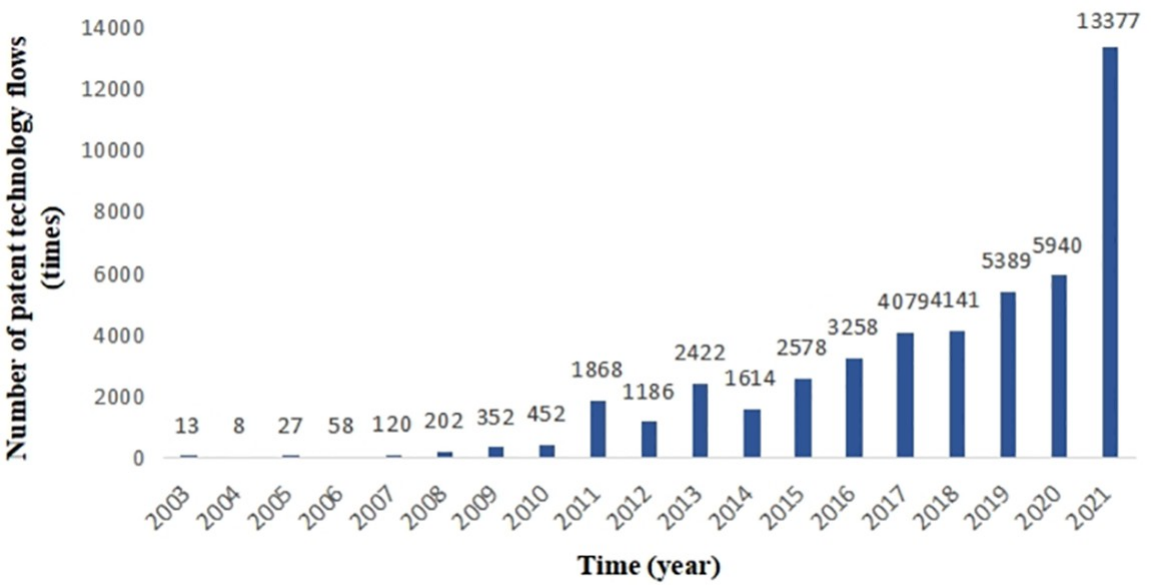
The number of patent technology flow in the Beijing-Tianjin-Hebei region in 2003–2021.

In the Beijing-Tianjin-Hebei region, there were 47,084 patent technology flows between 2003 and 2021. The number of patent technology flows grows together with the overall number of inventive patents over time. According to Fig 3, the number of patent technology flows in the Beijing-Tianjin-Hebei region over the study period had the following clear phase characteristics.

1. The number of patent flows in the Beijing-Tianjin-Hebei region was relatively low from 2003 to 2010, with a slow growth trend.
2. From 2011 to 2014, however, the number of patent flows significantly increased and displayed a large fluctuation trend, primarily because of The "Capital Economic Circle" concept, which was first introduced in the outline of the 12th Five-Year Plan in 2011 and encourages the integrated development of Beijing, Tianjin, and Hebei, may be the primary factor. The decline in patent technology flow in 2012 and 2014 may also be related to the chaos caused by patent infringement and counterfeiting during these two years.
3. The number of patent technology flows in the Beijing-Tianjin-Hebei region show a consistent rise between 2015 and 2020, which may be directly tied to the State Council’s decision in February 2014 to elevate the cooperative development of Beijing-Tianjin-Hebei to the status of a major national plan.
4. Since 2021 is the first year of the 14th Five-Year Plan period, there will be an increase in the number of patent technology flows in Beijing-Tianjin-Hebei, reaching more than twice the number of patent technology flows in 2020. Beijing, Tianjin, and Hebei released the "14th Five-Year Plan for Intellectual Property Development" in the first year of the 14th Five-Year Plan period, proposing to enhance the mechanism of collaborative development and promote suitable intellectual property flows, among other things.

**Fig 4:**
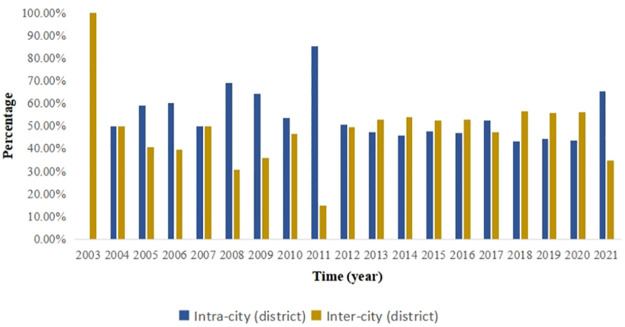
The Beijing-Tianjin-Hebei region’s share of the intra-city (district) and inter-city (district) technology flows in 2003–2021.

Based on the transfer (transferee) organization’s geographic and spatial situation, the intra-city (district) and inter-city (district) technology flows are differentiated. Fig 4 demonstrates that, with the exception of 2003, the patent technology flow in the Beijing-Tianjin-Hebei region was mostly carried out within cities (districts) prior to 2012. However, with the concepts of "Capital Economic Circle," "Beijing-Tianjin-Hebei Integrated Development," and "Beijing-Tianjin-Hebei Cooperative Development," the technology flow across administrative boundaries began. However, overall, in the Beijing-Tianjin-Hebei region, the intra-city (district) technology flow is greater than the inter-city (district) technology flow.

### 4.2 Subject characteristics

**Fig 5:**
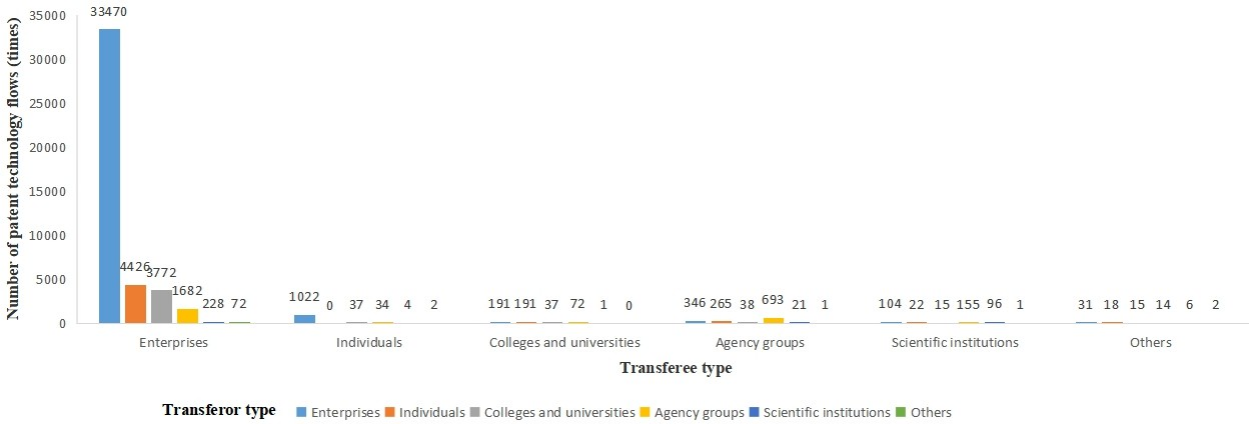
The amount of technology patents in the Beijing-Tianjin-Hebei region from 2003 to 2021, broken down by organization type.

This paper divides the transferring (transferee) parties in the flow of patent technology for inventions into six categories, including enterprises, individuals, colleges & universities, agency groups, scientific institutions, and others, where others represent types such as social organizations. Fig 5 shows the number of patent technology flow of each organization type in the Beijing-Tianjin-Hebei region. In the Beijing-Tianjin-Hebei region, enterprises are the primary driver of patent technology flow, and the number of patent technology flows in which enterprises participated from 2003 to 2021 accounts for 96.3% of the region’s overall patent technology flow. Specifically, the number of patent technology flows between enterprise → enterprises, individuals → enterprises, and colleges & universities → enterprises is in the top three of all flow combinations, which shows that enterprises have become the main body of patent technology flows in the Beijing-Tianjin-Hebei region, helping to narrow the gap between technology R&D and industrial landing. Colleges & Universities, and scientific institutions participate in less patent technology flows than individuals. The output of knowledge innovation is richer because more colleges & universities inventions have been granted patents than individual inventions have. It is clear that scientific institutions in the Beijing-Tianjin-Hebei region mostly pursue theoretical research and have not yet developed strong connections with the needs of business.

### 4.3 Spatial distribution characteristics

**Fig 6:**
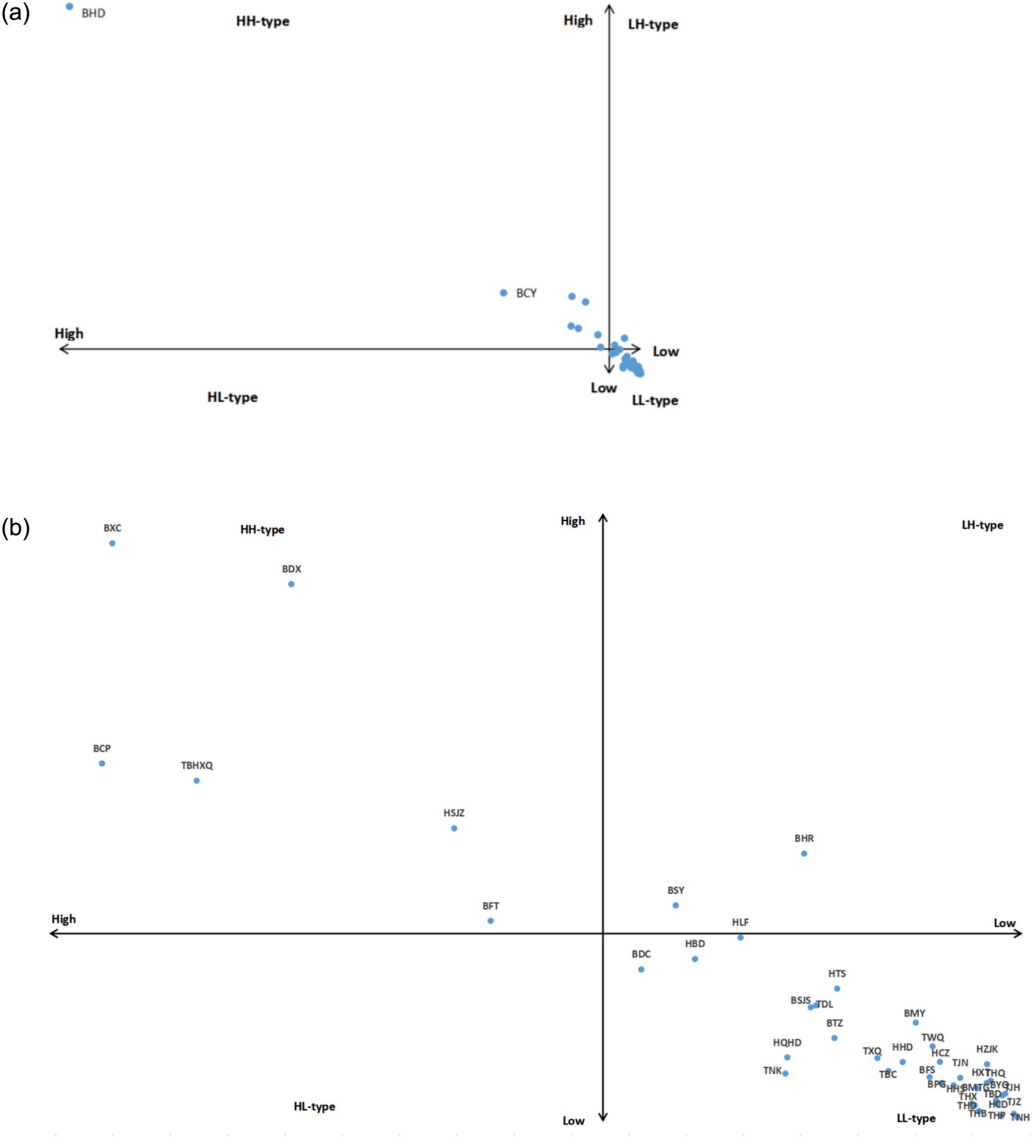
(a). Classification of patented technology flows by cities(districts) in the Beijing-Tianjin-Hebei region from 2003 to 2021. (b). Localized enlargement of the classification of patented technology flows in selected cities (districts) in the Beijing-Tianjin-Hebei region from 2003 to 2021.

In this paper, the 43 cities (districts) in Beijing-Tianjin-Hebei are divided into four types, i.e., HH-type of "high outflow and high inflow", HL-type of "high outflow and low inflow", LH-type with "low outflow and high inflow", and LL-type with "low outflow and low inflow" using the average value of the inflow and outflow of patented technology in each city (district) as the boundary, as shown in Fig 6(a), where the horizontal axis represents outflow and the vertical axis represents inflow. Since the distribution of cities (districts) in the LL-type is relatively dense, it is difficult to see the specific cities (districts), so the other parts of Fig 6(a) excluding BHD (Haidian District) and BCY (Chaoyang District) are partially enlarged, as shown in Fig 6(b).

From Fig 6(a) and 6(b), 43 cities (districts) are not involved in HL-type. There are significant differences in the number of patented technology flows among cities (districts) in the Beijing-Tianjin-Hebei region. The flow of patented technology is mainly concentrated in the central urban areas of Beijing, such as BHD (Haidian District),BCY (Chaoyang District) and BDX(Daxing District), while only HSJZ(Shijiazhuang) and TBHXQ(Binhai New Area) in Hebei and Tianjin have more frequent flows, showing a more obvious characteristic of "more in the central city and less in the peripheral cities". Specifically, the HH-type contains BCP (Changping District), BCY (Chaoyang District), BDX (Daxing District), BFT (Fengtai District), BHD (Haidian District), BXC (Xicheng District), TBHXQ (Binhai New Area), HSJZ (Shijiazhuang) eight cities (districts), which are more active in innovation and have stronger innovation capacity, and can realize continuous innovation through intra- or cross-regional technology flows. The LH-type contains two cities (districts), BHR (Huairou District) and BSY (Shunyi District), which are good at absorbing external knowledge, but their external spillover capacity needs to be improved. The remaining 33 cities (districts) belong to the LL-type, which have a low number of patented technology flows, lack of interaction between the region and the outside, and insufficient knowledge flows and spillovers.

## 5 Dynamic evolution of multi-level patent technology flow network in Beijing-Tianjin-Hebei region

### 5.1 Overall patent technology flow network

In order to more clearly reflect the dynamic characteristics of the patent technology flow network in the Beijing-Tianjin-Hebei region from 2003 to 2021, this paper divides the research time length into three periods: 2003–2009, 2010–2015, and 2016–2021. Among them, the network scale in 2003 is smaller, involving only four nodes. At the same time, to ensure the same time length in each period, this paper rounds off 2003 and adjusts the research time length to 2004–2009, 2010–2015, 2016–2021.

#### 5.1.1 Structural features

The patent technology flow network in Beijing-Tianjin-Hebei region consists of knowledge flows among organizations located in 43 cities (districts). In this paper, the network size, network density, average clustering coefficient and network transmission coefficient of the overall patent technology flow network in Beijing-Tianjin-Hebei region from 2004 to 2021 and in each period were measured using Ucinet software (Table 1) to reflect the structural characteristics of the overall patent technology flow network and its temporal changes.

**Table 1:** Temporal changes of structural indicators of patent technology flow network in Beijing-Tianjin-Hebei region in various periods.

Period	Network size	Network density	Average clustering coefficient	Network transmission coefficient
2004–2009	627	0.000935	0.003721	0.023256
2010–2015	4057	0.000165	0.013269	0.023123
2016–2021	12752	0.000057	0.013642	0.004824
2004–2021	15670	0.000048	0.018527	0.006238

The whole patent technology flow network in the Beijing-Tianjin-Hebei region has 627 nodes in 2004–2009, 4057 network nodes in 2010–2015, and 12752 network nodes in 2015–2021. In the process of the patent technology flow network going through three periods, many new flow subjects enter the network, and the network scale increases significantly. In terms of the network density of the patent technology flow network, with the expansion of the network scale, a large number of new flow subjects enter the network, and the network density gradually decreases, forming an increasingly loose and sparse network. As a whole, the patent technology flow network in Beijing-Tianjin-Hebei region has been in a low-density network state during the study period.

The average clustering coefficient increases significantly from the first to the third period, especially during the evolution from the first period (2004–2009) to the second period (2010–2015), the average clustering coefficient changes by a larger magnitude, and some of the network nodes become closer to each other, with a significant increase in the trend of small-group technology mobility. In contrast, the evolution from the second period (2010–2015) to the third period (2016–2021) is characterized by a smaller change in the average clustering coefficient and a slowdown in the growth of concentration among some network nodes. Although the size of the network is more than three times larger than the size of the network in the second period, fewer mobile subjects newly entering the network in the latter period are involved in the centralized network. As can be seen from Table 1, the network transmission coefficient gradually decreases from the first period to the third period, indicating that with the entry of a large number of new flow subjects, the existing nodes are more inclined to establish transferring relationships with the newly entered mobile subjects.

**Fig 7:**
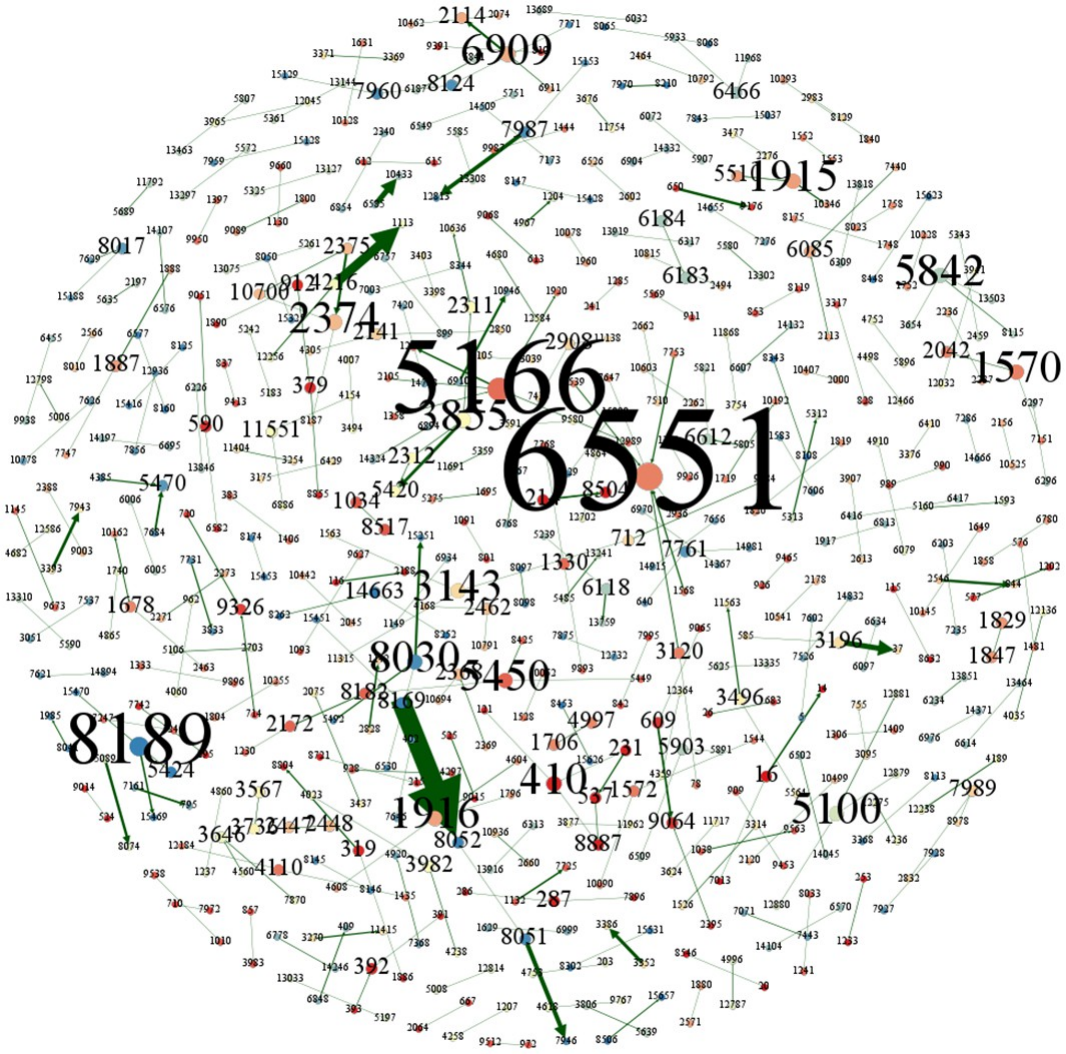
Overall patent technology flow network in Beijing-Tianjin-Hebei region, 2004–2009.

**Fig 8:**
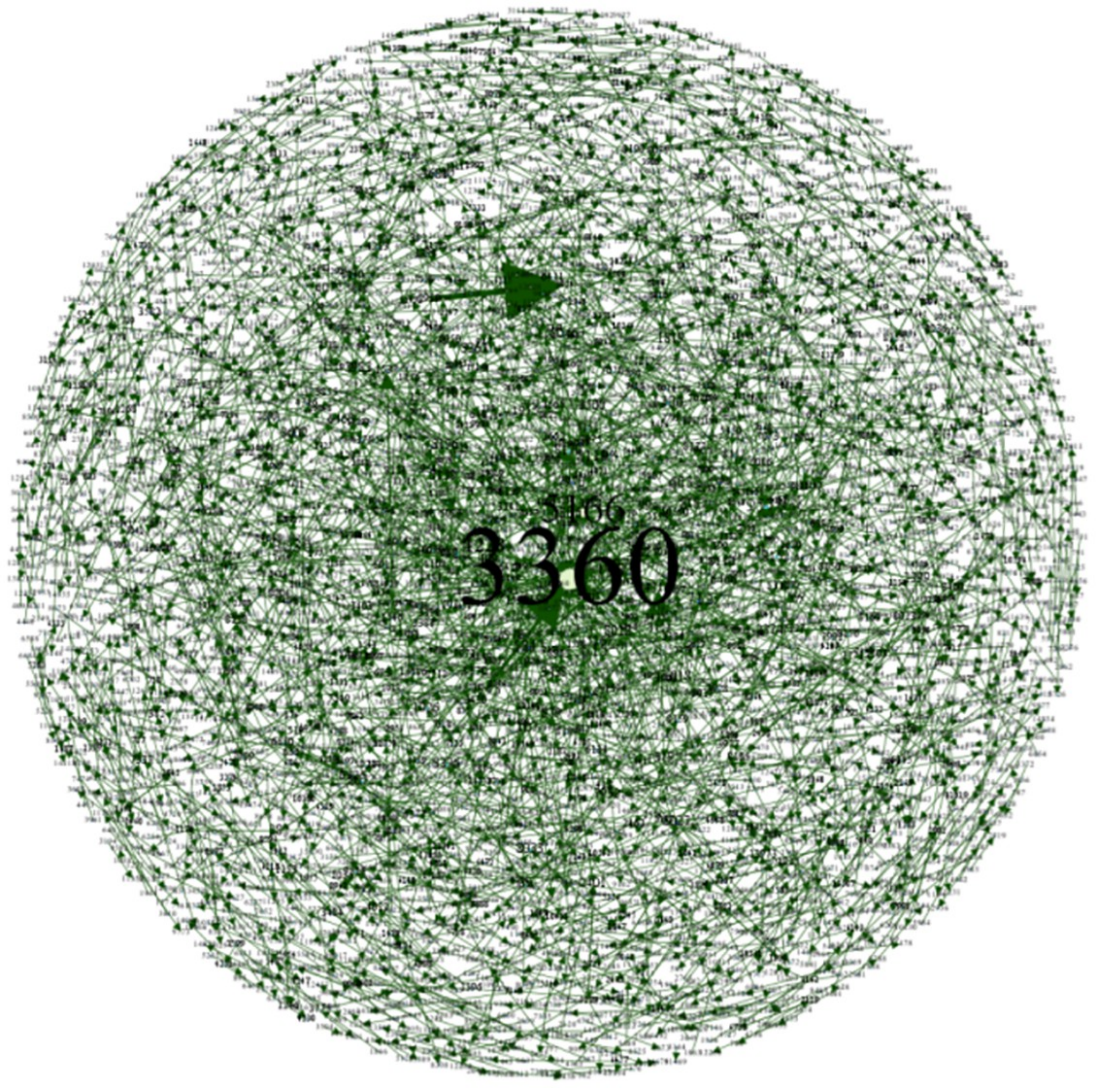
Overall patent technology flow network in Beijing-Tianjin-Hebei region, 2010–2015.

**Fig 9:**
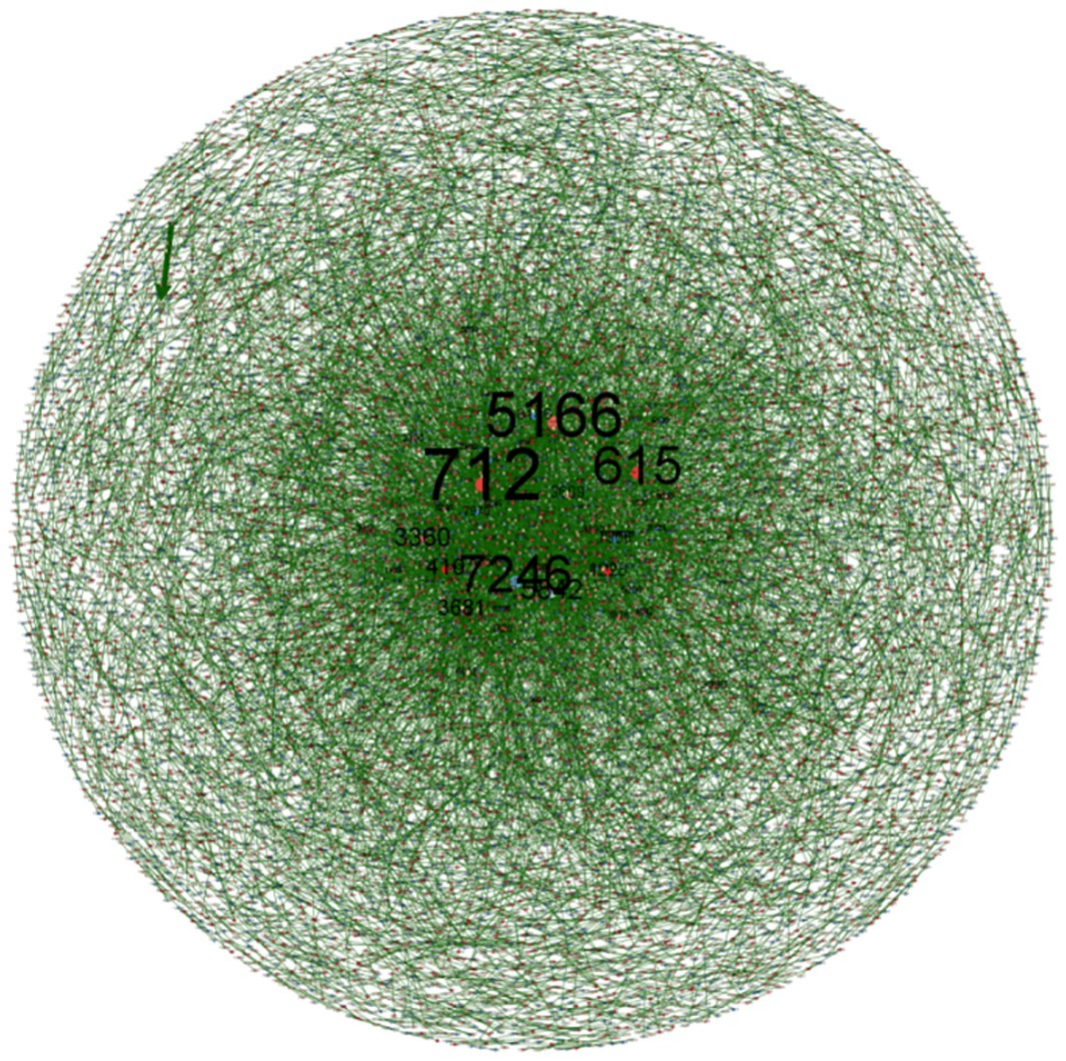
Overall patent technology flow network in Beijing-Tianjin-Hebei region, 2016–2021.

After measuring and analyzing the stage-by-stage structural characteristics of the overall patent technology flow network in the Beijing-Tianjin-Hebei region, this paper uses the Gephi 0.9.7 software to visualize the patent technology flow network, showing the patent technology flow network in the three periods of 2004–2009, 2010–2015, and 2016–2021, as respectively shown in Figs 7–9. Where the organizations are the nodes of the patent technology flow network, the size of the node circle represents the number of other organizations that directly transferor (transferee) technology with the node, i.e., the node degree, the number on the node represents the organization’s ID number in the study of this paper, and the line is the technology transfer relationship between organizations, and its thickness represents the number of times of technology transfers between organizations.

The main organizational nodes of the patent technology flow network in Beijing-Tianjin-Hebei region from 2004 to 2009 are Organization 6551 (Tianshili Pharmaceutical Group Co., Ltd.), Organization 5166 (Tsinghua University), Organization 8189 (China Banknote Printing and Minting Group Co., Ltd.), Organization 410 (Peking University), Organization 1570(Beijing Qin Tian Science & Technology Group Co., Ltd.), Organization 1915(Beijing SiFang Automation Co., Ltd.), Organization 1916(Beijing Sifang Rugang Concrete Products Co., Ltd), Organization 6909 (Wei Ming Bio-Agricultural Group Co., Ltd.), Organization 2374 (Increasepharm), Organization 3143(Feijiu Medical Technology (Beijing) Co., Ltd), etc. They have the highest number of transfer (transferee) relationships with other nodes and are among the top ten nodes in terms of node degree in the network of flow of patented technologies (Fig 7). In 2004–2009, there were more flows within the Beijing-Tianjin-Hebei region’s patent technology enterprise groups, which were mainly manifested in the transfer of the group’s subsidiaries and parent companies, as well as in the two ways of assigning individual job-related inventions to the enterprises. For example, the number of patented technology transfers from Organization 8169 (China Netcom) to Organization 8052 (China United Network Communications Group Co., Ltd.) was as high as 57. The number of patented technology transfers from Organization 4216 (BOE Technology Group Co., Ltd.) to Organization 1113 (Beijing BOE Optoelectronics Technology Co., Ltd.) reached 28. It can be seen that at this time, there is a clear trend of circulation within the main group in the Beijing-Tianjin-Hebei region, and there is insufficient spillover from the technology industry [44].

As can be seen in Fig 8, both the network nodes and the number of flows increased significantly in the second period (2010–2015), with the main organizational nodes being Organization 3360 (State Grid Co., Ltd.), Organization 5166 (Tsinghua University), Organization 1228 (University of Science and Technology Beijing), Organization 8030 (Institute of Computing Technology, Chinese Academy of Sciences), Organization 410 (Peking University), Organization 2395 (Beijing University of Posts and Telecommunications), Organization 9578 (China State Railway Group Corporation), Organization 615 (Beijing University of Technology), Organization 712 (Beihang University), Organization 7943 (China Electric Power Research Institute Co., Ltd.), etc. The flow of patented technologies within enterprise groups remains obvious. Among them, Organization 2989 (Datang Mobile Communications Equipment Co., Ltd.) made the highest number of patented technology transfers to Organization 3024 (Telecommunications Science and Technology Research Institute Co., Ltd.), amounting to 1,266. Unlike the previous period, the number of colleges and universities among the major nodes with node degrees in the top ten has risen, from two in the previous period to six in this period, while the number of patented technology transfers has also increased significantly.

As can be seen from Fig 9, in the third period (2016–2021), the main organizational nodes in the patented technology flow network in the Beijing-Tianjin-Hebei region are Organization 712 (Beihang University), Organization 5166 (Tsinghua University), Organization 615 (Beijing University of Technology), Organization 7246 (Yanshan University), Organization 5842 (Tianjin University), Organization 3360 (State Grid Co., Ltd.), Organization 410 (Peking University), Organization 3681 (Hebei University of Science and Technology), Organization 1228 (University of Science and Technology Beijing) and Organization 3609 (Hebei University of Science & Technology). Compared to the previous two periods, the number of colleges and universities in the top ten major nodes in terms of node degree has risen further, from six in the previous period to nine in this period, and the node degree of each node significantly increases. It shows that over time, the colleges and universities as the main producers of scientific and technological achievements, have gradually increased their participation in the Beijing-Tianjin-Hebei region’s patent technology flow network.

#### 5.1.2 Node characteristics

This paper uses Ucinet software to measure the weighted centrality, closeness centrality, and betweenness centrality of the nodes in the overall patented technology flow network in the Beijing-Tianjin-Hebei region from 2004 to 2021, and lists the organizations of the nodes that are ranked among the top ten in terms of the weighted out-degree centrality of the overall patented technology flow network during the three periods and their closeness centrality, and betweenness centrality respectively, as shown in Table 2. As a whole, the mean value of weighted centrality has been increasing over the three periods, and closeness centrality has continued to decrease. It suggests that organizations are participating in the flow of patented technologies more and more frequently over time, but that the interconnectedness of the organizations is weakening as the size of the network continues to grow. The top ten organizations in terms of weighted out-degree centrality have constantly changed across the periods, but Tsinghua University has been consistently ranked among them. Its mediated centrality has continued to rise, from 0.001 in the first period to 0.007 in the second period to 0.012 in the third period. It can be seen that Tsinghua University occupies a very important position in the Beijing-Tianjin-Hebei patented technology flow network and has a strong control over the flow of patented technology.

**Table 2:** Main node characteristics of patented technology flow network in Beijing-Tianjin-Hebei region in various periods.

Period	Organization Name	Weighted centrality		Closeness centrality		Betweenness centrality
		Weighted out-degree	Weighted in-degree	In-Closeness	Out-Closeness	
2004–2009	China Netcom	58	0	0.159	0.160	0.000
	BOE Technology Group Co. Ltd.	31	0	0.159	0.160	0.000
	Central Iron & Steel Research Institute Co.	17	0	0.159	0.160	0.000
	Tongfang Co., Ltd.	16	0	0.159	0.160	0.000
	China Unicom	14	0	0.159	0.160	0.000
	China Aerospace Times Electronics Co.	13	0	0.159	0.160	0.000
	Hebei Ealing Pharmaceutical Research Institute Co.	13	0	0.159	0.161	0.000
	Institute of Computing Technology, Chinese Academy of Sciences	12	0	0.159	0.160	0.000
	Tsinghua University	12	2	0.160	0.161	0.001
	Guodian Technology & Environment Group Co. Ltd.	10	0	0.159	0.160	0.000
2010–2015	Datang Mobile Communications Equipment Co.	1271	0	0.025	0.025	0.000
	China Electric Power Research Institute Co.	734	11	0.025	0.025	0.002
	Beijing BOE Optoelectronics Technology Co.	402	2	0.025	0.025	0.000
	Beijing Qizhi Business Consulting Co.	199	1	0.025	0.025	0.000
	Potevio Information Technology Co.	193	183	0.025	0.025	0.000
	China Potevio	182	193	0.025	0.025	0.000
	Tsinghua University	162	6	0.025	0.025	0.007
	Beijing Nonferrous Metals Research Institute Co.	121	0	0.025	0.025	0.000
	Peking University	95	3	0.025	0.025	0.002
	National Center for Nanoscience and Technology	83	4	0.025	0.025	0.000
2016–2021	Telecommunications Science and Technology Research Institute Co.	4331	0	0.008	0.008	0.000
	Beijing Nonferrous Metals Research Institute Co.	821	0	0.008	0.008	0.000
	Beijing Qihoo Technology Co.	613	30	0.008	0.008	0.000
	BAIC Foton Automotive Co. Ltd.	571	122	0.008	0.008	0.000
	Beihang University	483	5	0.008	0.008	0.001
	Beijing JD Shangke Information Technology Co.	471	9	0.008	0.008	0.000
	Beijing University of Technology	469	10	0.008	0.008	0.001
	Baidu Online Network Technology (Beijing) Co.	403	3	0.008	0.008	0.000
	China National Petroleum Corporation Bohai Drilling & Exploration Engineering Co.	371	0	0.008	0.008	0.000
	Tsinghua University	368	64	0.008	0.008	0.012

In order to clarify the roles played by different types of organizations in the overall patent technology flow network in the Beijing-Tianjin-Hebei region and the roles they play, this paper divides the types of organizations into six categories: enterprises, individuals, colleges & universities, agency groups, scientific institutions, and others, and analyzes the node characteristics of different types of organizations respectively. Table 3 is produced after categorizing and quantifying the weighted centrality, closeness centrality, and betweenness centrality of each type of organization node. Enterprises are the organizational types with the highest weighted centrality, closeness centrality, and betweenness centrality over the course of different time periods, while differences in centrality for other organizational types are less pronounced. This suggests that enterprises serve as the primary hubs of the patent technology flow network in the Beijing-Tianjin-Hebei region. Individual invention patent technology transfer also occupies a high proportion in the Beijing-Tianjin-Hebei region, mainly in the form of individual invention patents owned by enterprises. The amount of technical flows coming from colleges & universities has significantly increased, and their the out-closeness centrality is higher than in-closeness centrality, while the betweenness centrality is lower in comparison. This shows that colleges & universities have some radiation ability to other organization types in the patent technology flow network in the Beijing-Tianjin-Hebei region, but they do not currently play the role of "bridge." Even though agency groups and scientific institutions have fewer technology flows, their in-closeness centrality and betweenness centrality are better, indicating that they have some resource integration power in the Beijing-Tianjin-Hebei region’s patent technology flow network. Indicating that the patent technology flow in the Beijing-Tianjin-Hebei region is gradually active and no longer heavily relies on a single type of organization for patent technology transfer, the gap of intermediary centrality among the organization types gradually narrows over time.

**Table 3:** Characteristics of patent technology flow network nodes in Beijing-Tianjin-Hebei region by organization type.

Period	Organization type	Weighted centrality		Closeness centrality		Betweenness centrality
		Weighted in-degree	Weighted out-degree	In-Closeness	Out-Closeness	
2004–2009	Individuals	39	162	0.62500	0.71429	0.20000
	Enterprises	222	51	1.00000	0.83333	0.70000
	Colleges & Universities	7	33	0.55556	0.71429	0.00000
	Scientific institutions	3	4	0.55556	0.55556	0.00000
	Agency groups	8	29	0.83333	0.50000	0.00000
	Others	1	1	0.45455	0.55556	0.00000
2010–2015	Individuals	258	1308	0.71429	1.00000	0.01667
	Enterprises	2175	326	1.00000	1.00000	0.29167
	Colleges & Universities	101	589	0.71429	0.83333	0.00000
	Scientific institutions	16	37	0.83333	0.62500	0.00000
	Agency groups	74	320	0.83333	1.00000	0.09167
	Others	16	60	0.71429	0.55556	0.00000
2016–2021	Individuals	802	3439	1.00000	1.00000	0.01250
	Enterprises	7781	1317	1.00000	1.00000	0.01250
	Colleges & Universities	347	3255	0.83333	1.00000	0.00000
	Scientific institutions	267	219	1.00000	1.00000	0.01250
	Agency groups	589	1608	1.00000	1.00000	0.01250
	Others	67	15	1.00000	0.83333	0.00000
2004–2021	Individuals	1099	4909	1.00000	1.00000	0.01250
	Enterprises	10178	1694	1.00000	1.00000	0.01250
	Colleges & Universities	455	3877	0.83333	1.00000	0.00000
	Scientific institutions	286	260	1.00000	1.00000	0.01250
	Agency groups	671	1957	1.00000	1.00000	0.01250
	Others	84	76	1.00000	0.83333	0.00000

### 5.2 Dynamic evolution of the intra-city (district) patent technology flow network

When analyzing the network of patented technology flows of the intra-city (district), due to the large number of cities (districts) and limited space, some cities (districts) have smaller network sizes and simpler relationships between nodes. Therefore, this section only lists some cities (districts) as sample objects to describe the network of patented technology flows within their cities (districts). The majority of issued invention patents and intra-city (district) patent technology flow in the Beijing-Tianjin-Hebei region are produced in eight cities (districts), including BHD(Haidian District), BCY(Chaoyang District), HSJZ(Shijiazhuang), TBHXQ(Binhai New Area), BDX(Daxing District), BXC(Xicheng District), BCP(Changping District), and HBD(Baoding). Therefore, this paper firstly measures the structural characteristics of intra-city (district) patent technology flow network in eight cities (districts), and the specific results are shown in Table 4. On this basis, three cities (districts), namely, BHD, HSJZ and TBHXQ, are selected as the key cities (districts) to visualize and analyze their intra-city (district) patent technology flow network.

**Table 4:** Structural characteristics of intra-city (district) patent technology flow network of selected cities (districts).

City (District)	Period	Network size	Network density	Average clustering coefficient	Network transmission coefficient
BHD	2004–2009	160	0.003656	0.000000	0.000000
	2010–2015	730	0.000926	0.007479	0.013834
	2016–2021	1755	0.000413	0.012706	0.003340
	2004–2021	2356	0.000321	0.015799	0.004183
BCY	2004–2009	25	0.021667	0.000000	0.000000
	2010–2015	139	0.004171	0.000000	0.000000
	2016–2021	417	0.001539	0.004801	0.016892
	2004–2021	539	0.001200	0.005764	0.022222
HSJZ	2004–2009	25	0.025	0.000000	0.000000
	2010–2015	185	0.003437	0.038417	0.107143
	2016–2021	635	0.001066	0.012271	0.008689
	2004–2021	774	0.000896	0.017967	0.012484
TBHXQ	2004–2009	18	0.029412	0.000000	0.000000
	2010–2015	128	0.004798	0.034635	0.133333
	2016–2021	384	0.001659	0.019965	0.05
	2004–2021	478	0.001417	0.023745	0.044118
BDX	2004–2009	19	0.029240	0.000000	0.000000
	2010–2015	138	0.004073	0.023188	0.187500
	2016–2021	384	0.001598	0.015582	0.057143
	2004–2021	501	0.001242	0.017487	0.068627
BXC	2004–2009	15	0.047619	0.000000	0.000000
	2010–2015	61	0.011201	0.000000	0.000000
	2016–2021	147	0.004100	0.007370	0.033333
	2004–2021	199	0.003375	0.012603	0.019231
BCP	2004–2009	19	0.029240	0.000000	0.000000
	2010–2015	77	0.005566	0.011785	0.166667
	2016–2021	272	0.002388	0.018246	0.034483
	2004–2021	348	0.001896	0.020538	0.043689
HBD	2004–2009	4	0.166667	0.000000	0.000000
	2010–2015	48	0.015514	0.000000	0.000000
	2016–2021	259	0.002454	0.000000	0.000000
	2004–2021	294	0.002230	0.018424	0.019231

#### 5.2.1 Structural features

1. *2004–2009*. The structural characteristics of the intra-city (district) patent technology flow networks of the eight chosen cities (districts) varied noticeably between 2004 and 2009. The intra-city (district) patent technology flow network has 160 nodes in BHD, 25 in BCY and BDX, and less than 20 in the other five cities (districts). There are obvious differences in network size between the cities (districts), with BHD being about eight times larger than most cities (districts). Among the eight cities (districts), HBD’s intra-city (district) patent technology flow network is the smallest. Despite having the greatest intra-city (district) patent technology flow network, BHD’s network density is the lowest when compared to the other seven cities (districts). In general, cities (districts) with lower network size have relatively high network densities at this time. Numerous mobile subjects joined the network as a result of the network’s gradual development in size, but because they did not establish stronger connections, the network’s density gradually shrank. At this point, although the network sizes of BCY and HSJZ are the same, the network density of HSJZ is slightly higher than that of BCY, which shows that HSJZ is more closely connected among mobile subjects. The average clustering coefficient is used to gauge how closely connected the nodes in the patent technology flow network are to one another. The mobile subjects in these cities (districts) did not realize mutual connection in the intra-city (district) patent technology flow network during this period, and they had not yet formed the trend of small group technology flow, as evidenced by the average clustering coefficients of all eight cities (districts) during this period being 0. The network transmission coefficient shows whether there is a chance for transfer relationships to form between mobile subjects in the network and the transferee(transferor) of the transferor (transferee). The fact that none of the eight cities (districts) have any network transmission coefficients during this time period suggests that mobile subjects there are more likely to seek out new mobile subjects from outside the network for the transfer of patent technology than they are to establish transfer relations with the transferee(transferor) of the transferor (transferee).
2. *2010–2015*. The intra-city (district) patent technology flow network of each city (district) underwent more significant change between 2010 and 2015 compared to the first period. More flow subjects joined the patent technology flow network of each city (district), and the network’s size increased exponentially or by a factor of several. With 730 organizations becoming nodes of the intra-city (district) patent technology flow network, BHD continues to be the city (district) in the Beijing-Tianjin-Hebei region with the largest intra-city (district) patent technology flow network. HSJZ’s network size achieved a large jump with 185 nodes, surpassing BCY and ranking second among the eight cities (districts). Compared to the previous period, while the network size of the eight cities (districts) increased significantly, the network density decreased in all of them during this period. Except for BCY, BXC, and HBD, the average clustering coefficients and network transmission coefficients of the other five cities (districts) have increased. This indicates that the flow subjects in the network are more inclined to establish transferring relationships with the transferee(transferor) of the transferor (transferee), and that the tendency of the small-group technology flow has increased.
3. *2016–2021*. The intra-city (district) patent technology flow network of the eight cities (districts) is still undergoing substantial changes as the period 2016–2021 approaches. From the second period of 2010–2015 to the third period of 2016–2021, the network’s size dramatically increases, and an increasing number of flow subjects join the intra-city (district) patent technology flow network. BHD and BXC are the cities (districts) with the biggest and smallest network scales, respectively. The intra-city (district) patent technology flow network includes 1,755 flow subjects participating in BHD, compared to just 147 in BXC. Although there is a big variety in network scale between cities (districts), BXC has the highest network density and the closest connections between moving subjects. HSJZ, TBHXQ, and BDX’s average clustering coefficients have decreased, and the trend of small group technology flow has not improved. Except for BCY, BXC, and HBD, the network transmission coefficients of the other five cities (districts) have decreased.

**Fig 10:**
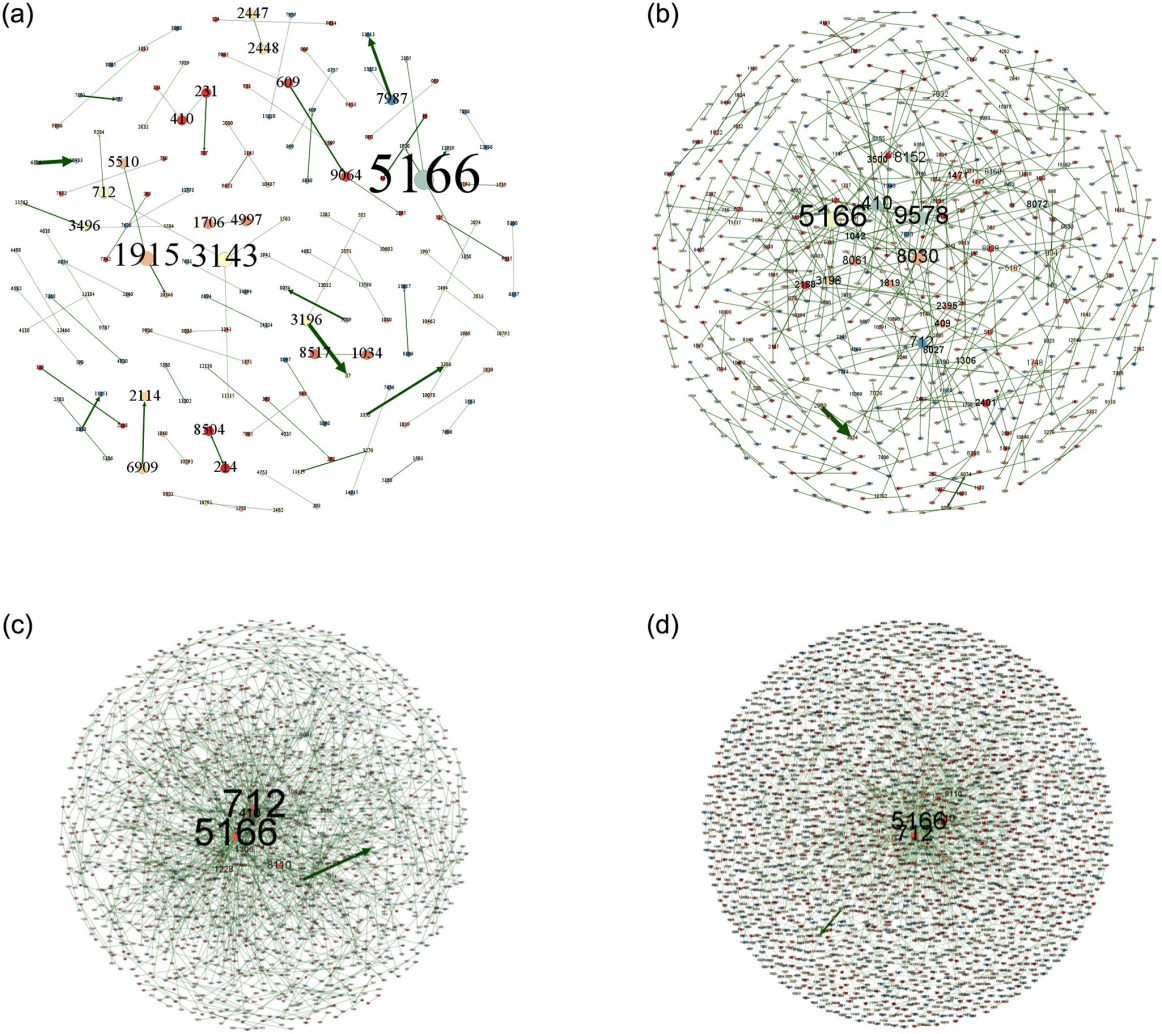
Intra-city (district) patent technology flow network in BHD, 2004–2021. a.2004-2009, b.2010-2015, c.2016-2021, d.2004-2021.

**Fig 11:**
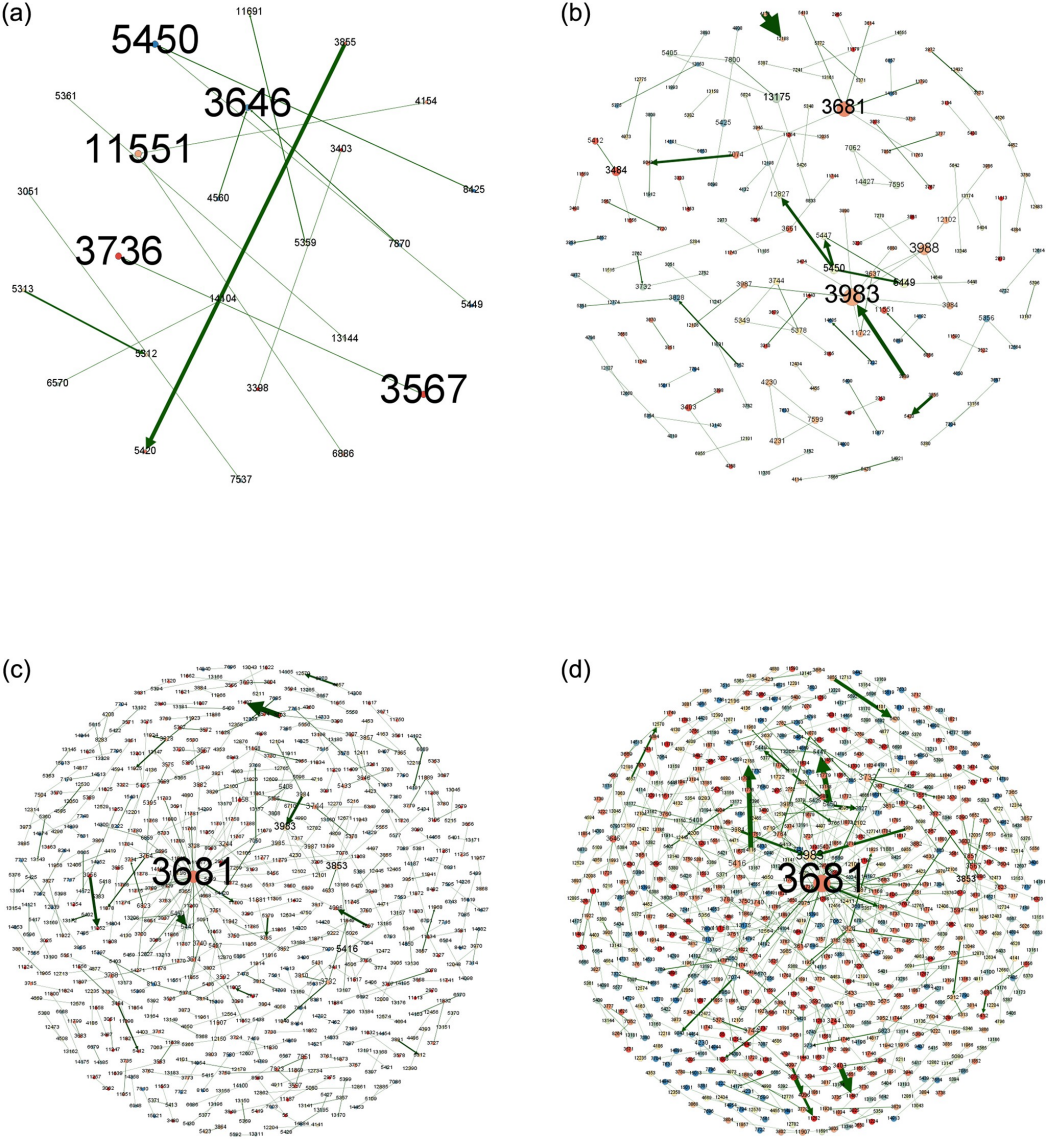
Intra-city (district) patent technology flow network in HSJZ, 2004–2021. a.2004-2009, b.2010-2015, c.2016-2021, d.2004-2021.

**Fig 12:**
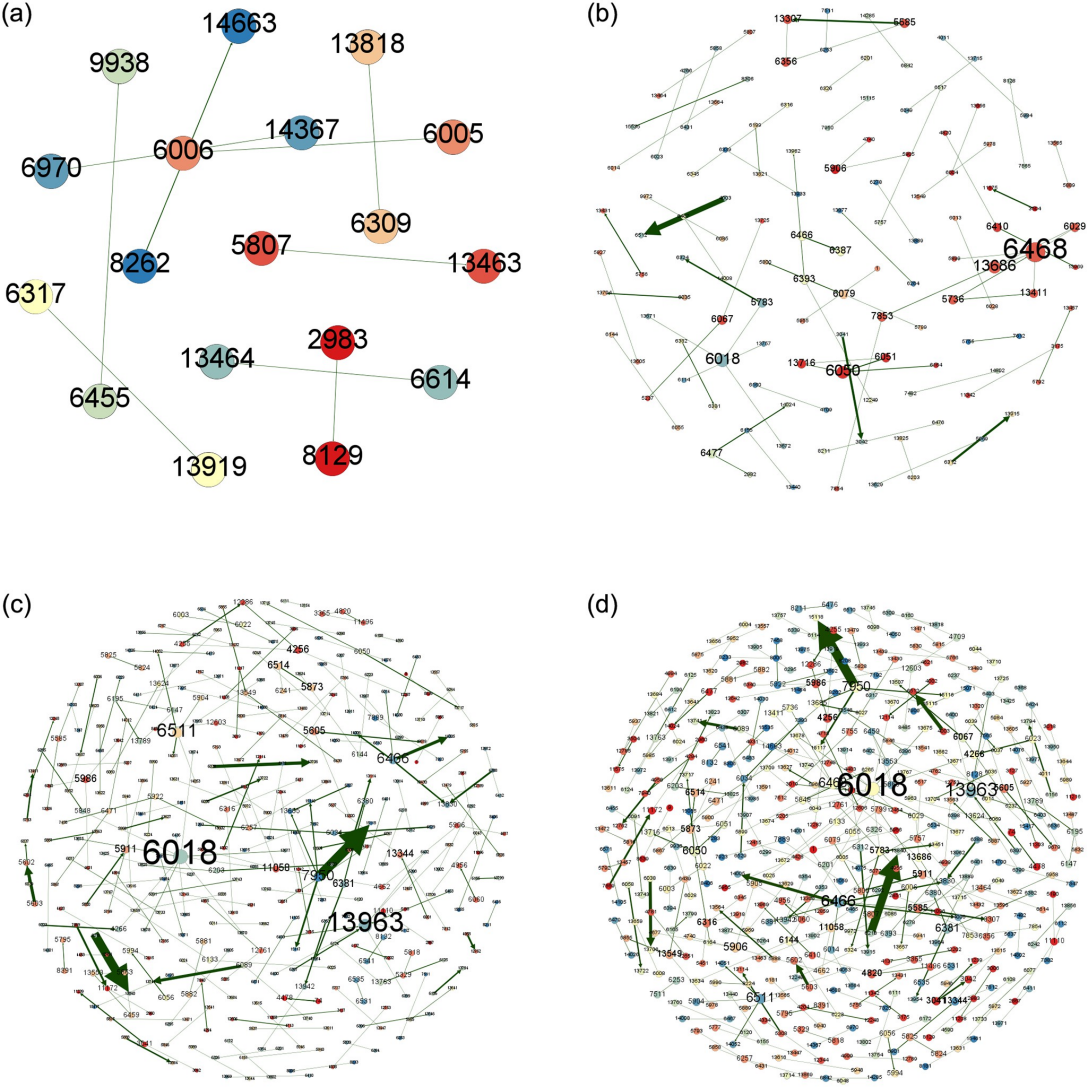
Intra-city (district) patent technology flow network in TBHXQ, 2004–2021. a.2004-2009, b.2010-2015, c.2016-2021, d.2004-2021.

In order to more clearly portray the intra-city (district) patent technology flow network of key cities (districts), this paper uses Gephi 0.9.7 software to visualize the intra-city (district) patent technology flow network, and shows the patent technology flow of BHD, HSJZ and TBHXQ from 2004–2009, 2010–2015, 2016–2021, and 2004–2021, as shown in Figs 10–12.

Organizations like Organization 5166 (Tsinghua University), Organization 712 (Beihang University), and Organization 410 (Peking University) serve as core nodes in the intra-city (district) patent technology flow network in BHD. Other nodes have created numerous patent technology transfer pairs outside of the clusters these core nodes have created, but they haven’t created a connectivity network. Several clusters with core nodes at Organization 3681 (Hebei University of Science and Technology) and Organization 3983 (North China Pharmaceutical Co., Ltd.) make up the intra-city (district) patent technology flow network in HSJZ. In the intra-city (district) patent technology flow network in HSJZ from 2010 to 2015, Organization 3983(North China Pharmaceutical Co., Ltd.) is the node with the most connections to other organizations when comparing different time periods. And from 2016 to 2021, Organization 3681(Hebei University of Science and Technology)’s patent technology transfer activities outpaced those of Organization 3983(North China Pharmaceutical Co., Ltd.), making it the node with the greatest number of connections to other businesses in the HSJZ intra-city (district) patent technology flow network. The Organization 6018 (Tianjin University of Science & Technology), Organization 6468 (Tianjin Yaoyu Biotechnology Co., Ltd.), and Organization 13963(Tiandy Digital Technology Co., Ltd.) are the most dominant nodal organizations in the intra-city (district) patent technology flow network of TBHXQ. The scope of their technology flow is significantly greater than that of other organizations, demonstrating their absolute central position and influence in the network.

#### 5.2.2 Node characteristics

This paper uses Ucinet software to measure the weighted centrality, closeness centrality, and betweenness centrality of each node in the intra-city (district) patent technology flow network of three key cities (districts), BHD, HSJZ, and TBHXQ, and lists the top ten node organizations in the intra-city (district) patent technology flow network of the three key cities (districts) in terms of weighted out-degree centrality, as well as their closeness centrality and betweenness centrality, the details of which are shown in Table 5.

**Table 5:** Main node characteristics of the intra-city (district) patent technology flow network in BHD, HSJZ and TBHXQ.

City (District)	Organization Name	Weighted centrality		Closeness centrality		Betweenness centrality
		Weighted out-degree	Weighted in-degree	In-Closeness	Out-Closeness	
BHD	Institute of Telecommunications Science and Technology Co.	4368	1266	0.042	0.043	0.000
	Datang Mobile Communication Equipment Co.	1271	4308	0.042	0.043	0.000
	Beijing JD Shangke Information Technology Co.	352	9	0.042	0.042	0.000
	Tsinghua University	300	37	0.043	0.043	0.041
	Beihang University	289	29	0.043	0.046	0.039
	Baidu Online Network Technology (Beijing) Co.	238	4	0.042	0.043	0.000
	Putian Information Technology Co.	203	182	0.042	0.043	0.000
	China Putian Information Industry Co.	182	201	0.042	0.043	0.000
	Institute of Feed Research of Chinese Academy of Agricultural Sciences	140	0	0.042	0.043	0.000
	Beijing Sifang Automation Co.	135	32	0.042	0.042	0.000
HSJZ	Shih Pharma Group Zhongqi Pharmaceutical Technology (Shijiazhuang) Co.	69	2	0.129	0.13	0.001
	Hebei University of Science & Technology	63	7	0.13	0.136	0.027
	Electric Power Scientific Research Institute of State Grid Hebei Electric Power Co.	39	3	0.13	0.13	0.001
	Jia Huiping	35	0	0.129	0.129	0.000
	Hebei Ealing Pharmaceutical Research Institute Co.	24	0	0.129	0.129	0.000
	Tunghsu Group	22	0	0.129	0.13	0.000
	North China Pharmaceutical Hebei Huamin Pharmaceutical Co.	20	3	0.13	0.131	0.001
	North China Pharmaceutical Group Preparation Co.	18	0	0.129	0.131	0.000
	Hebei Synergy Environmental Technology Co.	18	8	0.13	0.13	0.001
	Jikai Equipment Manufacturing Co.	15	1	0.13	0.129	0.000
TBHXQ	The Eighteenth Research Institute of China Electronics Technology Group Corporation	77	0	0.204	0.206	0.000
	Tianjin Huanuo Semiconductor Material Technology Co.	49	0	0.204	0.204	0.000
	Tianjin Pharmaceutical Research Institute Co.	38	4	0.205	0.206	0.004
	Huahe (Tianjin) New Technology Development Co.	22	0	0.204	0.204	0.000
	Tianjin Lizhong Alloy Group Co.	18	0	0.204	0.204	0.000
	Tai Chong (Tianjin) Heavy Equipment Technology Development Co.	17	2	0.204	0.204	0.000
	Tianjin 712 Communication & Broadcasting Co.	17	0	0.204	0.205	0.000
	Dingzheng Animal Pharmaceutical (Tianjin) Co.	15	1	0.204	0.205	0.000
	Tianjin Beiyang Baichuan Biotechnology Co.	14	1	0.205	0.205	0.002
	Tianjin University of Science & Technology	13	1	0.204	0.21	0.005

In the intra-city (district) patent technology flow network in BHD, the weighted centrality of the top 10 important node organizations in terms of weighted out-degree centrality has obvious differences, but the differences in closeness centrality and betweenness centrality are smaller. Among them, Tsinghua University and Beihang University perform more prominently, while the betweenness centrality of more nodal organizations is 0. It can be seen that the nodal organizations with higher weighted out-degree centrality are more likely to form patent technology transfer pairs and do not realize a larger influence range and stronger ability to connect other nodes in the network. The differences in weighted centrality, closeness centrality, and betweenness centrality of the top 10 important node organizations in the intra-city (district) patent technology flow network in HSJZ are smaller than those in BHD. Although the weighted out-degree centrality of Hebei University of Science and Technology is smaller than that of Shiba Pharmaceutical Group Zhongqi Pharmaceutical Technology (Shijiazhuang) Company Limited, it’s closeness centrality and betweenness centrality are significantly higher than those of the latter. It can be seen that Hebei University of Science and Technology occupies a very important position in the intra-city (district) patent technology flow network of HSJZ, and has a certain degree of resource integration, radiation, and control ability. For TBHXQ, the 18th Research Institute of China Electronics Technology Group Corporation has the best weighted out-degree centrality performance, which is located in the first place of each node organization, and Tianjin University of Science & Technology has the best out-closeness centrality and betweenness centrality performance.

Table 6 shows the weighted centrality, closeness centrality, and betweenness centrality of nodes of different organization types in eight cities (districts). In contrast, the weighted centrality of enterprises is significantly higher, while individuals, colleges and universities, scientific institutions, agency groups, and others are relatively smaller. The betweenness centrality of enterprises in TBHXQ, BDX, BXC, and BCP is higher than that of other cities (districts), which means that enterprises have more influence in the intra-city (district) patent technology flow network of these four cities (districts) and give full play to the role of "bridge." Although the weighted centrality of individuals in BCY is lower than that of enterprises, its betweenness centrality is significantly higher than that of other organization types, indicating that individuals have a certain resource integration ability and control in the intra-city (district) patent technology flow network in BCY. The betweenness centrality of each organization type in BHD and HSJZ is smaller and less concentrated than that of other cities (districts), indicating that the intra-city (district) patent technology flow network of these two cities (districts) is more active in each organization type and does not rely excessively on a single type of organization for patent technology transfer. For HBD, although the weighted centrality of colleges and universities is smaller compared with that of enterprises, the out-closeness centrality and betweenness centrality perform better, which shows that colleges and universities have certain radiation power and resource integration power in the intra-city (district) patent technology flow network of HBD.

**Table 6:** Characteristics of intra-city (district) patent technology flow network nodes in 8 cities (districts) by organization type.

City (District)	Organization type	Weighted centrality		Closeness centrality		Betweenness centrality
		Weighted in-degree	Weighted out-degree	In-Closeness	Out-Closeness	
BHD	Individuals	211	716	0.71429	1.00000	0.01667
	Enterprises	12363	10646	1.00000	1.00000	0.13333
	Colleges & Universities	165	945	0.71429	0.83333	0.00000
	Scientific institutions	182	117	1.00000	0.83333	0.06667
	Agency groups	527	986	0.83333	1.00000	0.03333
	Others	23	61	1.00000	0.62500	0.00000
BCY	Individuals	66	213	0.80000	1.00000	0.58333
	Enterprises	1539	1285	0.80000	0.57143	0.00000
	Colleges & Universities	17	45	0.50000	0.80000	0.00000
	Scientific institutions	35	36	0.57143	0.50000	0.00000
	Agency groups	44	122	0.80000	66.667	0.25000
	Others	0	0	0.00000	0.00000	0.00000
HSJZ	Individuals	40	361	0.45455	0.33333	0.05000
	Enterprises	996	613	0.50000	0.33333	0.12500
	Colleges & Universities	23	106	0.41667	0.31250	0.00000
	Scientific institutions	5	5	0.16667	0.16667	0.00000
	Agency groups	47	24	0.45455	0.31250	0.02500
	Others	0	2	0.16667	0.41667	0.00000
TBHXQ	Individuals	28	83	0.57143	0.50000	0.00000
	Enterprises	877	800	1.00000	0.50000	0.58333
	Colleges & Universities	7	13	0.66667	0.40000	0.00000
	Scientific institutions	0	0	0.00000	0.00000	0.00000
	Agency groups	4	16	0.66667	0.40000	0.00000
	Others	0	4	0.20000	0.57143	0.00000
BDX	Individuals	29	110	0.38462	0.29412	0.00000
	Enterprises	729	631	0.50000	0.33333	0.50000
	Colleges & Universities	0	8	0.16667	0.38462	0.00000
	Scientific institutions	0	10	0.16667	0.38462	0.00000
	Agency groups	1	1	0.38462	0.29412	0.00000
	Others	1	0	0.55556	0.16667	0.00000
BXC	Individuals	6	58	0.45455	0.62500	0.20000
	Enterprises	562	528	0.45455	1.00000	0.60000
	Colleges & Universities	1	2	0.35714	0.55556	0.00000
	Scientific institutions	1	0	0.50000	0.16667	0.00000
	Agency groups	33	17	0.41667	0.62500	0.00000
	Others	4	2	0.38462	0.45455	0.00000
BCP	Individuals	12	113	0.10000	0.44444	0.00000
	Enterprises	687	574	0.50000	0.50000	0.41667
	Colleges & Universities	26	37	0.44444	0.40000	0.00000
	Scientific institutions	3	0	0.66667	0.20000	0.00000
	Agency groups	0	4	0.20000	0.66667	0.00000
	Others	0	0	0.00000	0.00000	0.00000
HBD	Individuals	32	127	0.44444	0.80000	0.58333
	Enterprises	540	430	0.44444	0.50000	0.16667
	Colleges & Universities	5	21	0.40000	0.57143	0.25000
	Scientific institutions	0	0	0.00000	0.00000	0.00000
	Agency groups	1	2	0.36364	0.50000	0.00000
	Others	2	0	0.44444	0.20000	0.00000

### 5.3 Dynamic evolution of the inter-city (district) patent technology flow network

#### 5.3.1 Structural features

After portraying the overall patent technology flow network in the Beijing-Tianjin-Hebei region and the intra-city (district) patent technology flow network of each city (district), this subsection constructs the inter-city (district) patent technology flow network in the Beijing-Tianjin-Hebei region with each city (district) as the node and measures the relevant network indicators to reflect the structural characteristics of the inter-city (district) patent technology flow network, which are shown in Table 7. The network consists of 43 cities (districts) in the Beijing-Tianjin-Hebei region as nodes and their inter-city (district) patent technology transfer relationships. On the whole, the size of the inter-city (district) patent technology flow network in the Beijing-Tianjin-Hebei region has been expanding, and all 43 cities (districts) participate in the network. The network density, the average clustering coefficient, and the network transmission coefficient increase significantly. The patent technology flow between cities (districts) is gradually active, and the trend of small group technology flow is obvious.

**Table 7:** Structural characteristics of the inter-city (district) patent technology flow network in the Beijing-Tianjin-Hebei region.

Period	Network size	Network density	Average clustering coefficient	Network transmission coefficient
2004–2009	33	0.090 909	0.316 368	0.196 552
2010–2015	43	0.219 823	0.524 460	0.387 032
2016–2021	43	0.487 265	0.676 560	0.601 177
2004–2021	43	0.525 471	0.697 736	0.633 150

**Fig 13:**
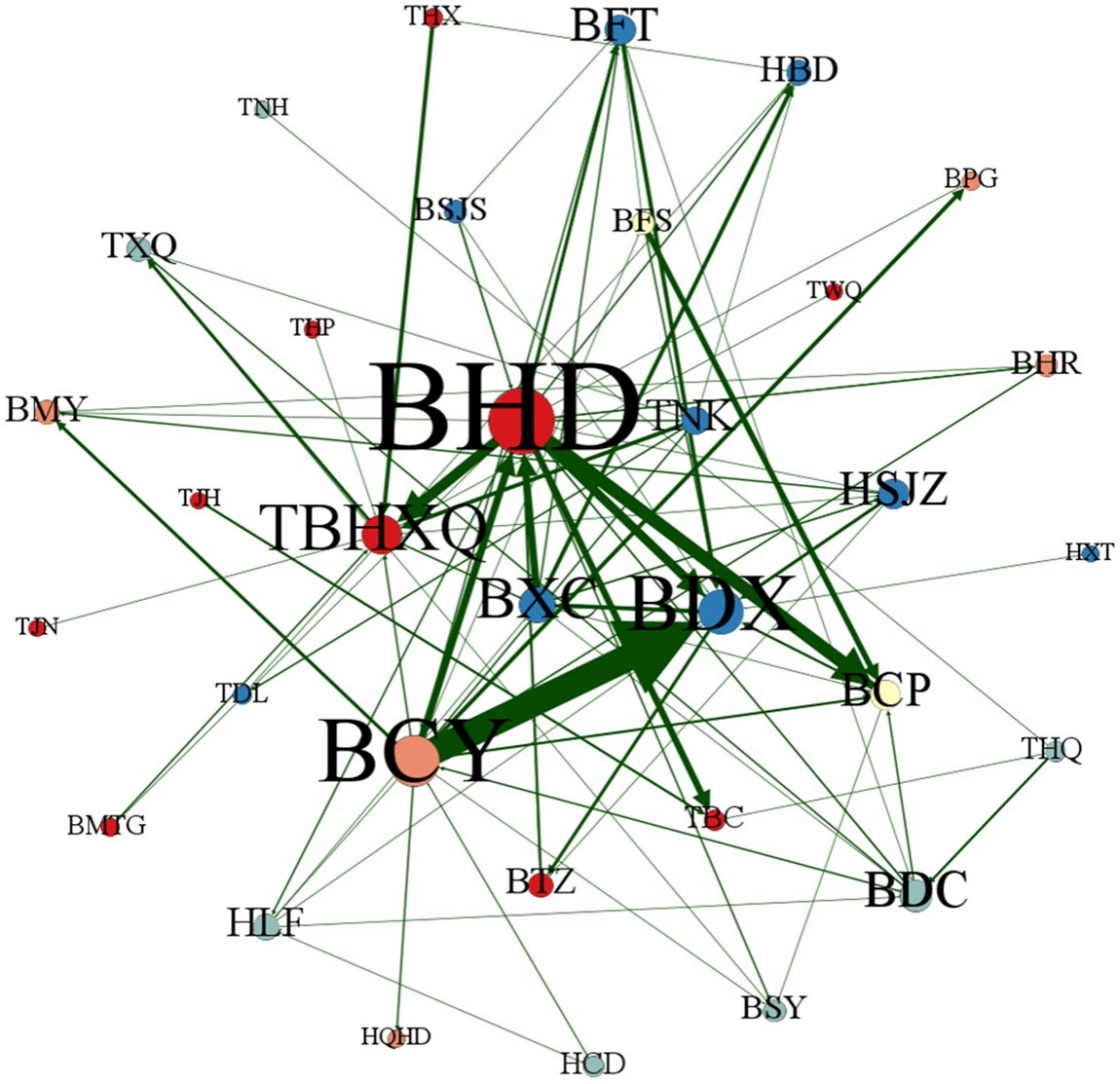
The inter-city (district) patent technology flow network in the Beijing-Tianjin-Hebei region, 2004–2009.

**Fig 14:**
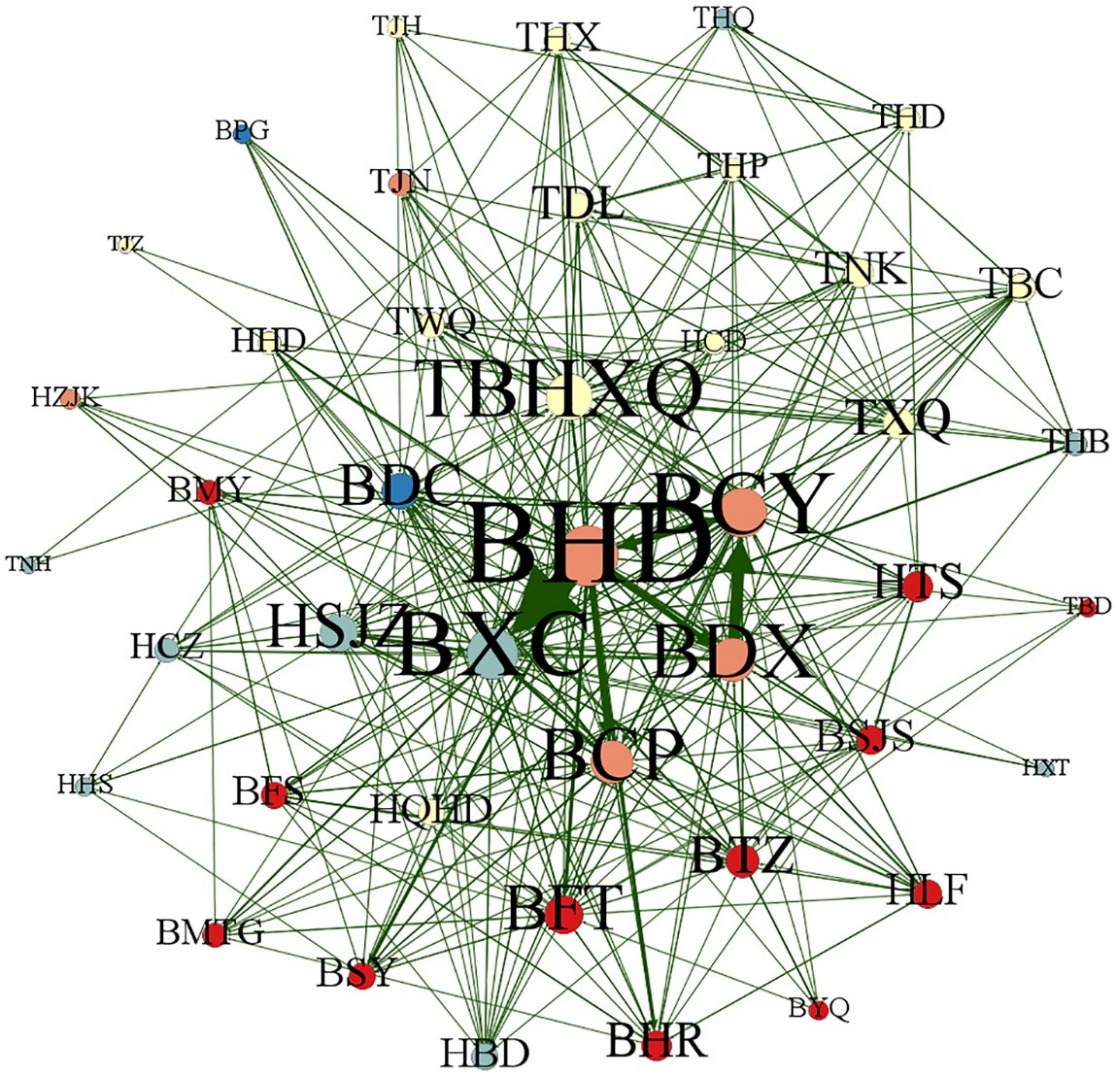
The inter-city (district) patent technology flow network in the Beijing-Tianjin-Hebei region, 2010–2015.

**Fig 15:**
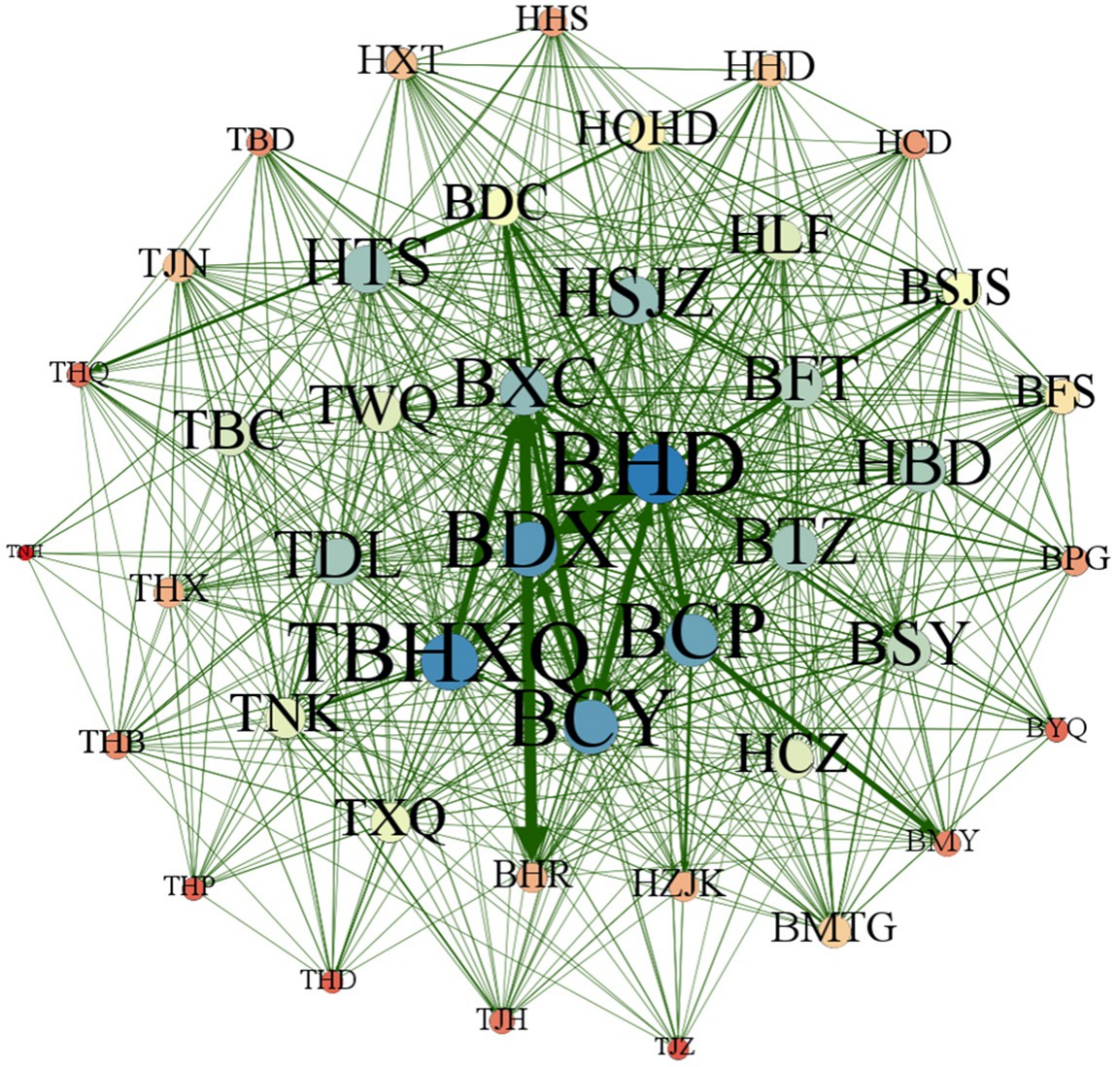
The inter-city (district) patent technology flow network in the Beijing-Tianjin-Hebei region, 2016–2021.

**Fig 16:**
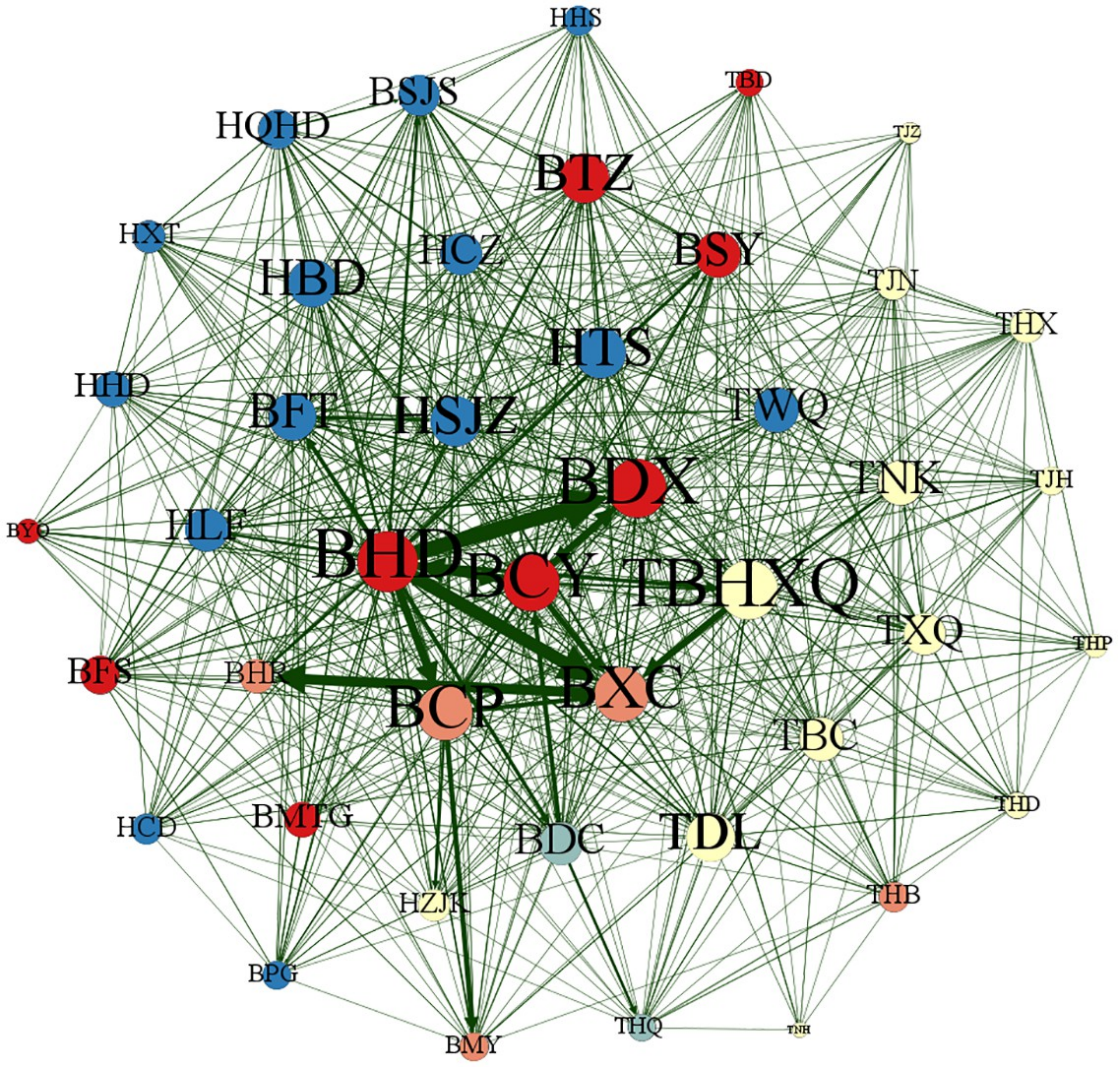
The inter-city (district) patent technology flow network in the Beijing-Tianjin-Hebei region, 2004–2021.

This section uses Gephi 0.9.7 software to map the patent technology flow network between cities (districts) in the Beijing-Tianjin-Hebei region in different periods, 2004–2009, 2010–2015, 2016–2021 and 2004–2021, to reflect more clearly the dynamic evolution of the patent technology flow network between cities (districts) in Beijing-Tianjin-Hebei region, as shown in Figs 13–16. Each city (district) is a node of the inter-city (district) patent technology flow network in the Beijing-Tianjin-Hebei region. The size of the node indicates the number of cities (districts) with which the node city (district) directly conducts patent technology transfer. The same color of nodes means they belong to the same cluster after clustering process. The connecting line is the patent technology transfer relationship between cities (districts), and its thickness represents the number of patent technology transfers between cities (districts).

1. *2004–2009*. During the period from 2004 to 2009, the inter-city (district) patent technology flow network in the Beijing-Tianjin-Hebei region was relatively sparse. Only 33 cities (districts) participated in the inter-city (district) patent technology flow network. BYQ(Yanqing District), TBD(Baodi District), THB(Hebei District), THD(Hedong District), TJZ(Jizhou District), HCZ(Cangzhou), HHD(Handan), HHS(Hengshui), HTS(Tangshan), and HZJK(Zhangjiakou) did not enter the inter-city (district) patent technology flow network at this time. It means that these ten cities (districts) and the other 33 cities (districts) are not engaged in patent technology flow and are still in a relatively isolated state. The network density of the inter-city (district) patent technology flow network is only 0.090 909, the average clustering coefficient is 0.316 368, and the transmission coefficient is 0.196 552. BHD is the core node of the inter-city (district) patent technology flow network in the Beijing-Tianjin-Hebei region at this time and has the closest technology flow with TBHXQ and BCP. From the color of the nodes, BHD, BTZ(Tongzhou District), BMTG(Mentougou District), TBHXQ, TBC(Beichen District), TJN(Jinnan District), TWQ(Wuqing District), and TJH(Jinghai District), etc. have the same color and belong to the same cluster. BCY, BMY(Miyun District), BHR(Huairou District), BPG(Pinggu District), and HQHD(Qinhuangdao), etc. belong to the same cluster. BCP and BFS(Fangshan District) belong to the same cluster. BSY(Shunyi District), THQ(Hongqiao District), TNH(Ninghe District), TXQ(Xiqing District), HCD(Chengde), HLF(Langfang), and BDC(District), etc. belong to the same cluster. HSJZ, HBD, HXT(Xingtai), BFT(Fengtai District), BSJS(Shijingshan District), BXC, and BDX, etc. belong to the same cluster. From the cluster perspective, the inter-city (district) patent technology flow network in the Beijing-Tianjin-Hebei region shows specific preferences. Each city (district) prefers to conduct patent technology transfer with its own province (city) or neighboring cities (districts). Geographical proximity has a greater influence on the network structure of the inter-city (district) patent technology flow network in the Beijing-Tianjin-Hebei region.
2. *2010–2015*. With the concept of "Capital Economic Circle" put forward, the integrated development of Beijing, Tianjin and Hebei continues to advance. In the period of 2010–2015, the inter-city (district) patent technology flow network in the Beijing-Tianjin-Hebei region became more intensive compared with the previous period. The size of the network reached 43. Cities (districts) that did not join the patent technology flow network in the previous period began to establish links with other cities (districts). The network density in this period is 0.219 823, which is higher than that of the previous period. It can be seen from Fig 15 that the patent technology transfer relationship increased significantly at this time. The average clustering coefficient increased from 0.316 368 to 0.524 460, and the flow subjects in each city (district) began to show the trend of small group mobility and formed clusters. BHD is still the most influential node in the inter-city (district) patent technology flow network in the Beijing-Tianjin-Hebei region. Meanwhile, the influence of BXC has increased. During this period, BHD, BXC, BCY, BDX and other cities (districts) became nodes with frequent patent technology flow. In terms of the color of nodes, the clustering situation in this period has changed somewhat compared with the previous period. However, on the whole, cities (districts) still tend to transfer patent technology with their own province (city) or neighboring cities (districts).
3. *2016–2021*. In the period of 2016–2021, the size of the inter-city (district) patent technology flow network has included all the cities (districts) in the Beijing-Tianjin-Hebei region, and more patent technology transfer relationships have been generated in the network, as shown in Fig 15. The network density becomes 0.487 265, which is a more obvious increase compared with the previous two periods. It implies a significant increase in the number of organizations and relationships involved in cross-regional patent technology transfer. The average clustering coefficient and network transmission coefficient have also increased to a certain extent. BHD has been in the most central position in the inter-city (district) patent technology flow network in the Beijing-Tianjin-Hebei region. The gap between the size of nodes in the network of TBHXQ, BDX, BCY, BCP, BXC, HSJZ, and other cities (districts) and BHD is gradually narrowing, and the influence in the network is gradually increasing. In terms of the color of the nodes, the clustering situation in this period has increased the cohesion among cities (districts) compared with the previous period. Patent technology transfer in this period is mainly influenced by the geographical distance between cities (districts).

The characteristics of the inter-city (district) patent technology flow network in the Beijing-Tianjin-Hebei region for the whole period from 2004 to 2021 remain consistent with those of the 2016–2021 period. All 43 cities (districts) participate in the inter-city (district) patent technology flow network for the whole period. The network density, average clustering coefficient, and network transmission coefficient do not differ significantly compared with those of 2016–2021. BHD is always the most frequent region in the Beijing-Tianjin-Hebei region in terms of patent technology flow with other cities (districts).

#### 5.3.2 Node characteristics

In this paper, on the basis of drawing the inter-city (district) patent technology flow network in the Beijing-Tianjin-Hebei region, the position of each city (district) in the inter-city (district) patent technology flow network is determined by calculating the characteristic indicators of weighted centrality, closeness centrality, and betweenness centrality of each city (district), and the results are shown in Table 8, which are analyzed as follows.

1. In terms of weighted centrality, the weighted centrality of BHD has remained stable at the top in all periods. The weighted out-degree centrality is significantly higher than the weighted in-degree centrality, indicating the absolute central position and influence of BHD in the inter-city (district) patent technology flow network in the Beijing-Tianjin-Hebei region. BHD has a strong influence on the flow of patented technology to other cities (districts), and is able to obtain considerable benefits from the flow of patented technology from other cities (districts). The weighted centrality of BXC has increased during 2010–2015 and is in the second place among 43 cities (districts), mainly due to the frequent patent technology flow between the research units and subsidiaries of State Grid Corporation. Specifically, for each city in Hebei Province, the weighted in-degree centrality is significantly higher than the weighted out-degree centrality for all 10 cities except HQHD during 2016–2021. It indicates that there is a certain innovation gap among cities in Hebei Province, and central cities such as HSJZ and HBD have limited patent technology flow to other cities, and more advanced technological innovation results are obtained from some regions in Beijing and Tianjin. For Tianjin, the phenomenon of polarization of patent technology flow in each district is serious. With significant differences, TBHXQ has been in the forefront at all stages, while TNH and TJZ have been at the bottom of the cities (districts) in Beijing-Tianjin-Hebei region.
2. In terms of closeness centrality, the closeness centrality of each city (district) from 2016 to 2021 has increased significantly compared with the previous two periods, which means that each city (district) has improved both in terms of integration and radiation, and the inter-city (district) patent technology flow network in the Beijing-Tianjin-Hebei region has formed a better foundation and has certain long-term development momentum. BHD, TBHXQ, and BDX have the highest in-closeness and out-closeness centrality, and the out-closeness centrality is greater than the in-closeness centrality, which means that these three regions have stronger radiation ability to other cities (districts) in the inter-city (district) patent technology flow network in the Beijing-Tianjin-Hebei region. HTS’s in-closeness centrality leaped from 2016 to 2021, ranking 3rd among 43 cities (districts), thanks to its active connection to Beijing-Tianjin innovation sources in recent years, and its efforts to integrate innovation resources and promote the transfer and transformation of scientific and technological achievements. Lower out-closeness centrality, such as TNH, TJZ, BMY, and BYQ, these areas have more scarce innovation resources, innovation achievements, sparse innovation relationships, and lower technology flow benefits.
3. In terms of betweenness centrality, the betweenness centrality of each city (district) fluctuated greatly in these three stages. The betweenness centrality of cities (districts) with high weighted centrality or closeness centrality, represented by BHD, BCY, and TBHXQ, has shown a continuous downward trend during the study period, and the gap between their betweenness centrality and that of other cities (districts) has gradually narrowed. It indicates that over time, the 43 cities (districts) in the Beijing-Tianjin-Hebei region have gradually become more active in the flow of patented technologies, and are no longer overly dependent on a particular city (district) for the transfer of patented technologies. In addition, the betweenness centrality of HTS, TBC, and TDL gradually increased during the study period and ranked among the top 10 cities (districts) in the 43 cities (districts) from 2016 to 2021, which shows that they play the role of "bridge" in the inter-city (district) patent technology flow network in the Beijing-Tianjin-Hebei region.

**Table 8:** Main node characteristics of the inter-city (district) patent technology flow network in the Beijing-Tianjin-Hebei region.

Period	City (District)	Weighted centrality		Closeness centrality		Betweenness centrality
		Weighted in-degree	Weighted out-degree	In-Closeness	Out-Closeness	
2004–2009	BCP	29	13	0.11034	0.12598	0.00671
	BCY	16	64	0.11470	0.13333	0.19040
	BDX	66	15	0.11470	0.12851	0.07103
	BDC	6	9	0.10774	0.12903	0.03929
	BFS	1	10	0.10596	0.12030	0.00000
	BFT	10	8	0.10997	0.12648	0.00951
	BHD	42	73	0.11470	0.13734	0.24608
	BHR	3	3	0.10631	0.12598	0.00428
	BMTG	1	2	0.10631	0.11268	0.00000
	BMY	7	4	0.11034	0.12167	0.00731
	BPG	6	0	0.12075	0.03030	0.00000
	BSJS	1	4	0.10561	0.12598	0.00000
	BSY	5	0	0.12500	0.03030	0.00000
	BTZ	6	4	0.10959	0.12549	0.00000
	BXC	8	25	0.10997	0.12955	0.03146
	BYQ	0	0	0.00000	0.00000	0.00000
	TBD	0	0	0.00000	0.00000	0.00000
	TBC	12	0	0.13445	0.03030	0.00000
	TBHXQ	32	7	0.11594	0.12261	0.15449
	TDL	2	1	0.10458	0.10667	0.00706
	THB	0	0	0.00000	0.00000	0.00000
	THD	0	0	0.00000	0.00000	0.00000
	THP	0	1	0.03030	0.12549	0.00000
	THX	1	5	0.10191	0.11307	0.00504
	THQ	0	5	0.03030	0.15385	0.00000
	TJZ	0	0	0.00000	0.00000	0.00000
	TJN	0	1	0.03030	0.12549	0.00000
	TJH	0	3	0.03030	0.03125	0.00000
	TNK	5	9	0.11307	0.12549	0.11148
	TNH	1	0	0.03125	0.03030	0.00000
	TWQ	1	0	0.11896	0.03030	0.00000
	TXQ	6	3	0.10922	0.11765	0.00902
	HBD	8	2	0.10922	0.11594	0.0494
	HCZ	0	0	0.00000	0.00000	0.00000
	HCD	2	1	0.10631	0.12075	0.00141
	HHD	0	0	0.00000	0.00000	0.00000
	HHS	0	0	0.00000	0.00000	0.00000
	HLF	3	4	0.10847	0.12308	0.00763
	HQHD	2	0	0.11765	0.03030	0.00000
	HSJZ	4	9	0.11228	0.12698	0.07765
	HTS	0	0	0.00000	0.00000	0.00000
	HXT	0	1	0.03030	0.13169	0.00000
	HZJK	0	0	0.00000	0.00000	0.00000
2010–2015	BCP	363	304	0.40385	0.60000	0.03586
	BCY	628	498	0.39252	0.73684	0.07847
	BDX	289	523	0.38182	0.66667	0.05865
	BDC	139	70	0.35897	0.61765	0.02124
	BFS	19	48	0.33333	0.5122	0.00272
	BFT	176	81	0.36842	0.62687	0.02664
	BHD	503	1941	0.42424	0.82353	0.19077
	BHR	125	18	0.36842	0.51220	0.00491
	BMTG	31	18	0.35593	0.48276	0.00110
	BMY	28	10	0.35000	0.49412	0.00319
	BPG	9	25	0.31111	0.48837	0.00000
	BSJS	50	45	0.33071	0.58333	0.00574
	BSY	136	24	0.35294	0.50602	0.00990
	BTZ	106	48	0.36842	0.55263	0.01409
	BXC	1163	236	0.42857	0.64615	0.15506
	BYQ	17	4	0.32061	0.47727	0.00050
	TBD	6	1	0.32061	0.40000	0.00000
	TBC	47	30	0.35000	0.53846	0.01814
	TBHXQ	142	109	0.38889	0.66667	0.12181
	TDL	68	26	0.36207	0.53165	0.01605
	THB	7	68	0.34146	0.49412	0.01033
	THD	16	22	0.32308	0.50602	0.00814
	THP	8	14	0.31343	0.53165	0.00300
	THX	14	30	0.34711	0.55263	0.01388
	THQ	6	13	0.28378	0.47191	0.00361
	TJZ	1	1	0.28378	0.41176	0.00010
	TJN	47	5	0.35000	0.42424	0.00376
	TJH	15	6	0.30882	0.46154	0.00210
	TNK	26	57	0.34426	0.57534	0.00942
	TNH	4	0	0.40385	0.02326	0.00000
	TWQ	42	5	0.36842	0.49412	0.01169
	TXQ	39	32	0.35593	0.56757	0.02409
	HBD	26	18	0.36207	0.50000	0.00306
	HCZ	18	19	0.34146	0.50000	0.00496
	HCD	22	4	0.33871	0.49412	0.00262
	HHD	19	10	0.35000	0.49412	0.00217
	HHS	3	9	0.31343	0.44681	0.00047
	HLF	80	21	0.35294	0.53165	0.00150
	HQHD	13	17	0.35294	0.53846	0.00493
	HSJZ	65	109	0.37500	0.60000	0.04949
	HTS	34	48	0.36207	0.56000	0.01285
	HXT	6	1	0.31818	0.40000	0.00000
	HZJK	16	4	0.33871	0.43299	0.00086
2016–2021	BCP	962	1357	0.73684	0.91304	0.03430
	BCY	1174	2447	0.75000	0.93333	0.03430
	BDX	2012	610	0.80769	0.87500	0.03980
	BDC	566	741	0.62687	0.72414	0.00906
	BFS	146	91	0.63636	0.65625	0.00468
	BFT	426	753	0.68852	0.77778	0.01415
	BHD	1877	4151	0.87500	0.93333	0.04971
	BHR	1010	127	0.60000	0.61765	0.00250
	BMTG	120	56	0.60870	0.64615	0.00368
	BMY	489	212	0.60870	0.56000	0.00216
	BPG	194	52	0.58333	0.60870	0.00187
	BSJS	458	347	0.64615	0.68852	0.00699
	BSY	492	255	0.72414	0.72414	0.01402
	BTZ	268	332	0.76364	0.73684	0.01848
	BXC	1586	1505	0.76364	0.77778	0.02395
	BYQ	123	35	0.56757	0.56757	0.00029
	TBD	63	28	0.60870	0.57534	0.00155
	TBC	73	156	0.65625	0.75000	0.03267
	TBHXQ	904	1133	0.84000	0.89362	0.04713
	TDL	303	208	0.77778	0.72414	0.02790
	THB	52	42	0.60870	0.57534	0.00450
	THD	66	88	0.54545	0.59155	0.00072
	THP	21	37	0.55263	0.57534	0.00194
	THX	41	48	0.60870	0.60870	0.00583
	THQ	230	62	0.55263	0.6000	0.00285
	TJZ	35	11	0.58333	0.53846	0.00057
	TJN	111	59	0.64615	0.60000	0.00489
	TJH	134	21	0.60000	0.56000	0.00169
	TNK	163	466	0.63636	0.75000	0.01610
	TNH	18	10	0.54545	0.47191	0.00007
	TWQ	268	102	0.73684	0.65625	0.00966
	TXQ	190	208	0.66667	0.70000	0.01449
	HBD	333	255	0.73684	0.75000	0.01503
	HCZ	172	38	0.77778	0.62687	0.01251
	HCD	58	19	0.63636	0.56757	0.00379
	HHD	83	57	0.64615	0.60870	0.00717
	HHS	59	21	0.62687	0.58333	0.00215
	HLF	411	133	0.71186	0.67742	0.00842
	HQHD	70	307	0.63636	0.67742	0.00343
	HSJZ	527	254	0.77778	0.75000	0.01880
	HTS	388	83	0.82353	0.70000	0.03278
	HXT	69	58	0.64615	0.60000	0.00212
	HZJK	252	22	0.61765	0.60870	0.00369

## 6 Conclusions and suggestions

In order to illustrate the spatial and temporal evolutionary characteristics of the technology flow network in the Beijing-Tianjin-Hebei region of China, this research investigates the transfer of innovation patents in the region of Beijing-Tianjin-Hebei from 2003 to 2021. First, the quantitative changes, subject type, and spatial distribution of the patent technology flow in the Beijing-Tianjin-Hebei region are examined. Second, a multi-level patent technology flow network that included the overall network, the intra-city (district) network with organizations as nodes, and the inter-city (district) network with cities (districts) as nodes were built, and the structural characteristics and node characteristics of each level network were analyzed with special attention. The main findings are as follows.

First, in terms of the change in the number of flows, the number of flows of patented technologies has been growing over time, showing a clear phase characteristic over the study period. In the Beijing-Tianjin-Hebei region, the number of patent flows was relatively low from 2003 to 2010, with a modest growth tendency. Between 2011 and 2014, the number of patent flows in the Beijing-Tianjin-Hebei region started to increase noticeably and shown a major volatility tendency. With the synergistic development of Beijing-Tianjin-Hebei rising as a major national strategy, the number of patent flows in the Beijing-Tianjin-Hebei region from 2015 to 2021 shows an obvious and steady upward trend. On the whole, the intra-city (district) technology flow in the Beijing-Tianjin-Hebei region is higher than the inter-city (district) technology flow.

Second, in terms of the type of subject, enterprises are the main force of patent technology flow in the Beijing-Tianjin-Hebei region, and the number of patent technology flow involving enterprises accounts for 96.3% of the total patent technology flow in Beijing-Tianjin-Hebei region from 2003 to 2021. In contrast, the number of patent technology flows involving colleges and universities, and scientific institutions is lower than that of individuals.

Third, in terms of spatial distribution characteristics, there are significant differences in the number of patented technology flows among cities (districts) in the Beijing-Tianjin-Hebei region. 43 cities (districts) did not address HL-type. 76.74% of the cities (districts) belong to the LL-type, they have a low number of patented technology flows, lack of intra-regional and external interactions, and insufficient knowledge flows and spillovers. The flow of patented technology is mainly concentrated in the central urban areas of Beijing, such as BHD, BCY, and BDX, while only TBHXQ and HSJZ in Tianjin and Hebei have more frequent flows, showing a more obvious characteristic of "more in the central city and less in the peripheral cities".

Fourth, the overall patent technology flow network, intra-city (district) patent technology flow network, and inter-city (district) patent technology flow network in Beijing-Tianjin-Hebei region exhibit dynamic characteristics in the three periods of 2004–2009, 2010–2015, and 2016–2021, and the size of their networks all increase significantly. With the passage of time, more and more flow subjects participate in the patent technology flow network at the three levels. The network density of the overall patent technology flow network in Beijing-Tianjin-Hebei region decreases with the expansion of scale. The average clustering coefficient gradually increases. Some network nodes become closer to each other, and the trend of small group technology flow increases significantly. Enterprises are the core hub of the patent technology flow network in Beijing-Tianjin-Hebei region. Technology transfer of individual invention patents also occupies a high proportion in the Beijing-Tianjin-Hebei region, mainly in the form of individual invention patents owned by enterprises, and the participation of colleges and universities in the patent technology flow network in the Beijing-Tianjin-Hebei region gradually increases actively.

Fifth, BHD is the most developed intra-city (district) patent technology flow network among the 43 cities (districts) in the Beijing-Tianjin-Hebei region. Compared with BHD and TBHXQ, HSJZ has a more obvious phenomenon of polarization of its intra-city (district) patent technology flow network. The nodes of the intra-city (district) patent technology flow network have certain differences in weighted centrality, closeness centrality, and betweenness centrality. Enterprises in the TBHXQ, BDX, BXC, and BCP, the city (district) within the flow of patented technology network with greater influence, giving full play to the role of the "bridge". Individuals have a certain resource integration ability and control in the intra-city (district) patent technology flow network in BCY. Each organization type is more active in the intra-city (district) patent technology flow network of BHD and HSJZ. And does not rely excessively on a single type of organization for patent technology transfer. Colleges and universities have certain radiation power and resource integration power in the intra-city (district) patent technology flow network of HBD.

Sixth, at present, all 43 cities (districts) in the Beijing-Tianjin-Hebei region have established regional external links and channels for extra-regional knowledge exchange, which together constitute the inter-city (district) patent technology flow network. BHD, BCP, BDX, BCY, BXC, TBHXQ, HSJZ and other cities (districts) have a greater influence in the inter-city (district) patent technology flow network. With the passage of time, 43 cities (districts) in the Beijing-Tianjin-Hebei region have gradually become active in patent technology flow and no longer rely excessively on a certain city (district) for patent technology transfer.

These findings have certain theoretical significance. In the research process, this paper systematically reveals the evolution of the structural characteristics of the patent technology flow network between cities (districts) in the Beijing-Tianjin-Hebei region of China. From constructing a multi-level patent technology flow network to studying network characteristics, and then characterizing spatiotemporal evolution laws, it explores new research paths for the study of patent technology flow across urban (district) boundaries. The relevant research results have certain academic reference significance for enriching and improving the current research on cross-city (district) technology flow networks.

Based on the above findings, this paper proposes the following countermeasures:

1. A platform for the exchange, transfer, and transformation of patented technologies in the Beijing-Tianjin-Hebei region should be set up to reduce the transaction costs of cross-regional patented technologies, enhance the flow of cross-regional patented technologies, and break the current status quo, which is mainly based on the transfer of patented technologies among innovation subjects within the regions of various cities (districts). Based on actively releasing the scientific research potential of Beijing and improving the technology undertaking and transformation capacity of Tianjin and Hebei, strengthen the patent technology flow from the core cities (districts) to other marginal cities (districts) for patent technology transfer from all over the world, improve the radiation and diffusion capacity of patent technology transfer from the core cities (districts), and form a multi-level, multi-regional and all-round patent technology flow pattern of Beijing, Tianjin and Hebei.
2. The process of adjusting the technological and industrial layout of the Beijing-Tianjin-Hebei region should be accelerated to alleviate the current situation of high homogeneity of high-quality technologies in Tianjin and Hebei, and the mismatch between the supply of innovation in Beijing and the demand for innovation in Tianjin and Hebei. On the one hand, give full play to Beijing’s core leading role, relying on Beijing’s excellent universities and research institutes to carry out key technology and common technology research and development according to the actual technology needs of Tianjin and Hebei, on the basis of which Tianjin and Hebei will transform and reinvent key technology and common technology to improve their own innovation level. On the other hand, promote Beijing, Tianjin, and Hebei to jointly build a technology cooperation R&D platform, explore the establishment of innovation resources and innovation revenue sharing mechanism, improve the intellectual property protection system, and help the reasonable flow of technology elements in Beijing, Tianjin, and Hebei.
3. The regulations on patent technology transfer in each city (district) should be improved, and an effective institutional mechanism and incentive mechanism should be established, so as to actively encourage and guide the innovation main bodies within the region to establish cross-regional patent technology transfer relations and obtain more heterogeneous knowledge, while at the same time, optimizing the technological innovation environment within the region, promoting the flow and diffusion of such heterogeneous knowledge in the region, and enhancing the performance of regional innovation.

Although this paper employs a social network analysis approach to provide some valuable insights into the spatio-temporal evolution of technology flows in the Beijing-Tianjin-Hebei region of China, it also has some limitations. For example, the use of invention patent transfer data to construct intra-city (district) and inter-city (district) patent technology flow networks at the city (district) level as a reflection of formal technology flow relationships may ignore the informal technology flow processes among innovation agents. Therefore, the understanding and knowledge of the composition and structural features in the informal technology flow network can be further deepened in the future.

## Supporting information

S1 File (XLSX)
